# Microfluidic chip for precise trapping of single cells and temporal analysis of signaling dynamics

**DOI:** 10.1038/s44172-022-00019-2

**Published:** 2022-07-25

**Authors:** Nidhi Sinha, Haowen Yang, David Janse, Luc Hendriks, Ulfert Rand, Hansjörg Hauser, Mario Köster, Frans N. van de Vosse, Tom F. A. de Greef, Jurjen Tel

**Affiliations:** 1 Laboratory of Immunoengineering, Department of Biomedical Engineering, TU Eindhoven, 5600 MB Eindhoven, Netherlands; 2 Institute of Complex Molecular Systems, TU Eindhoven, 5600 MB Eindhoven, Netherlands; 3 Model Systems for Infection and Immunity, Helmholtz Centre for Infection Research, 38124 Braunschweig, Germany; 4 Cardiovascular Biomechanics Group, Department of Biomedical Engineering, TU Eindhoven, 5600 MB Eindhoven, Netherlands; 5 Computational Biology Group, Department of Biomedical Engineering, TU Eindhoven, 5600 MB Eindhoven, Netherlands

**Keywords:** Lab-on-a-chip, Cell biology

## Abstract

Microfluidic designs are versatile examples of technology miniaturisation that find their applications in various cell biology research, especially to investigate the influence of environmental signals on cellular response dynamics. Multicellular systems operate in intricate cellular microenvironments where environmental signals govern well-orchestrated and robust responses, the understanding of which can be realized with integrated microfluidic systems. In this study, we present a fully automated and integrated microfluidic chip that can deliver input signals to single and isolated suspension or adherent cells in a precisely controlled manner. In respective analyses of different single cell types, we observe, in real-time, the temporal dynamics of caspase 3 activation during DMSO-induced apoptosis in single cancer cells (K562) and the translocation of STAT-1 triggered by interferon γ (IFNγ) in single fibroblasts (NIH3T3). Our investigations establish the employment of our versatile microfluidic system in probing temporal single cell signaling networks where alternations in outputs uncover signal processing mechanisms.

## Abstract (web summary)

Nidhi Sinha, Haowen Yang and colleagues report a microfluidic large-scale integration chip to probe temporal single-cell signalling networks via the delivery of patterns of input signalling molecules. The researchers use their device to investigate drug-induced cancer cell apoptosis and single cell transcription (STAT-1) protein signalling dynamics.

## Introduction

The encoding and decoding of signals by cells from their cellular microenvironment is a tightly regulated process that simultaneously filters signal fluctuations or noise in the vicinity. The complexity of the cellular microenvironment constantly subjects the cells to proteins, molecules, and molecular patterns from pathogens to activate signaling pathways and define cellular responses^1–4^. Signaling molecules such as growth factors and immune stimulators are often released and modulated as a function of time and dose to precisely regulate cellular functions and prevent over activation^5^. The cells encode these signals and process temporally modulated information to influence downstream responses, but the underlying mechanism requires further understanding^6^. Comprehension of such well-regulated systems presents itself with the necessity to alter traditional methods of research and integration of technology that provides cells with the much-needed controlled microenvironment^7^.

Studies in the field of biology are often performed using traditional bulk methods, but recent years have witnessed a paradigm shift in the use of single-cell technologies owing to the inquisitiveness to understand cellular functions at the base level^8–10^. Single-cell technologies have helped researchers to recognize some of the most basic intercellular functions that often get masked with bulk methodologies^11,12^. One popular single-cell tool is microfluidics which can be combined with flow cytometry, mass cytometry, or microscopy for downstream analysis^13–16^. Microfluidic systems provide high control and precision over artificial cellular microenvironments and allow the replication of biological systems, in vitro, with high accuracy using small sample volume^17^. Also, lab-on-a-chip and micro total analytical systems (µTAS) have contributed towards the miniaturization of entire experimental workflow, on-chip^18^.

Microfluidic single-cell research is performed by isolating individual cells in channels or droplets to decode temporal activation and response dynamics of signaling pathways and functions by negating the effects of paracrine communication from the neighboring cells^19,20^. Furthermore, cell-pairing, to understand cellular communication and cytotoxicity at single-cell level in a noise-free environment, in which the cells are not subjected to paracrine communication or signal variations, is also feasible on microfluidic platforms^21^. There are primarily three broad categories of microfluidic designs that allow us to perform biological research at the level of single cells namely microfluidic devices with hydrodynamically formed arrays, droplet-based microfluidics, and microfluidic large-scale integration (mLSI) chips with integrated arrays^22^. While the first two allow for high-throughput analysis with simple design parameters, these two design categories are limited by the ability to provide controlled environmental conditions to the cells and delivery of complex stimuli patterns^23–25^. Droplet microfluidic platforms are not easily applicable for adherent cells, and arrays do not warrant isolated environment for single cells. More importantly, mLSI designs possess the unique ability to allow the delivery of time and dose-modulated inputs to multiple and individual cells cultured on the chip, making them a promising research tool in several labs^19^. mLSI chips are two-layer designs integrating several pneumatic membrane valves arranged as multiplexers for control of fluid flow and automation of experimental workflows^26–28^. Over the years mLSI chips have contributed vastly towards various biological applications, making them a promising and versatile approach^29–31^.

In this research, we have derived a simplified design of mLSI chips that will allow biological researchers to implement complex biological assays. Researchers can use this design to comprehend the influence of signal fluctuations, as a function of dose and time, on single-cell sensing. Additionally, in this setup, we can negate additional influences from the neighboring cells to investigate signaling dynamics with single-cell resolution. We accomplished this by integrating microfluidic channels with pillar-like structures, to physically isolate and trap cells and provide individual cells with a noise-free environment.

**Fig. 1:**
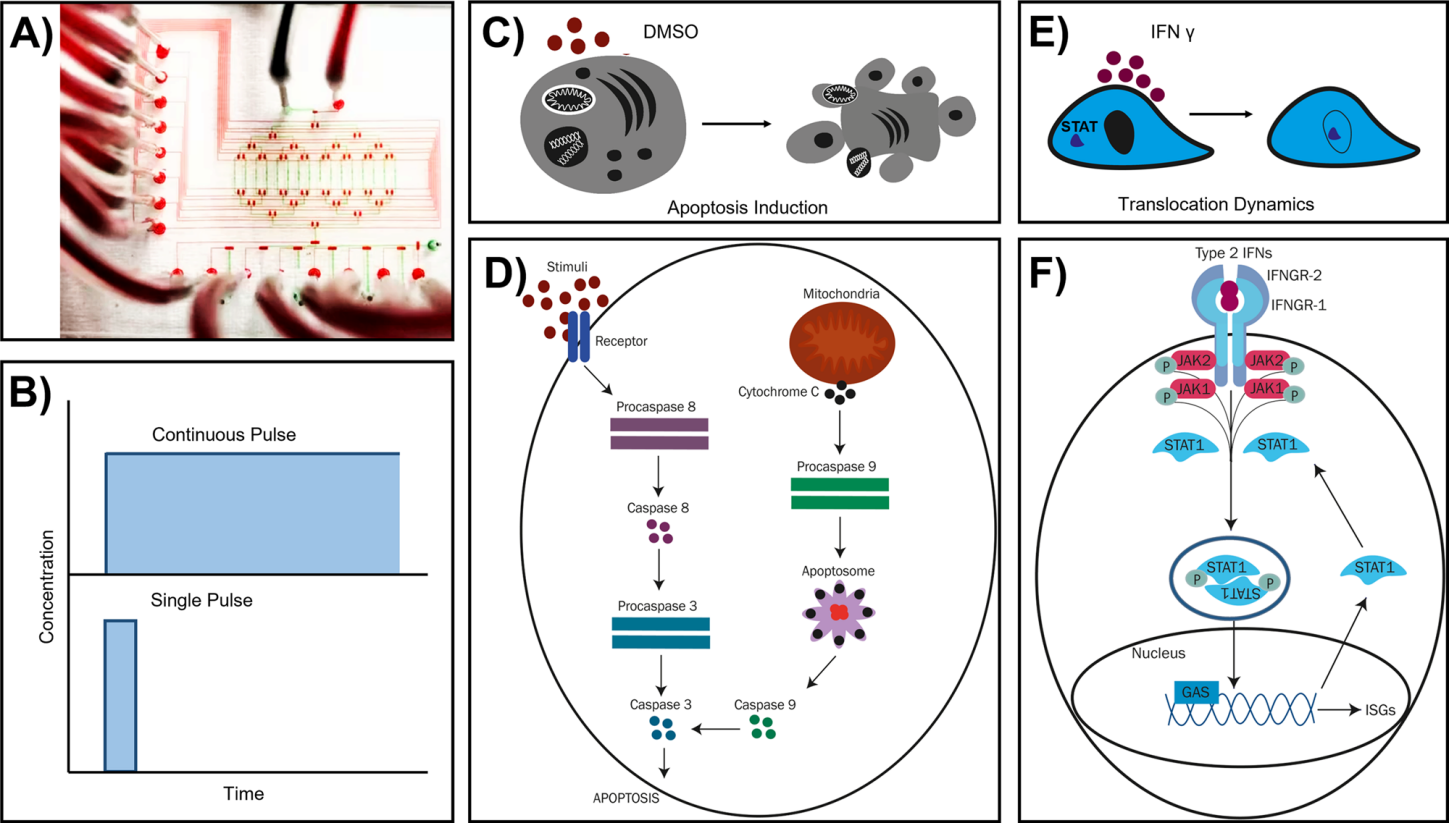
Objective of the research presented in this paper. **A** Two-layer microfluidic chip design to (**B**) deliver time- and dose-modulated input stimuli to single cells and investigate (**C**, **D**) apoptosis in K562 cell, activated upon treatment with different doses and time of DMSO and (**E**, **F**) the translocation dynamics of STAT-1 signaling protein in NIH3T3 cells, when stimulated with type 2 interferons.

We used this design to observe the timeline of drug-induced apoptosis in single K562 (chronic myelogenous leukemia) cells and the translocation dynamics of signal transducer and activator of transcription (STAT) protein from the cytoplasm to nucleus in murine fibroblast NIH3T3 cells when activated with type 2 interferons (Fig. 1). In this study, we delivered the stimuli as a pulsatile function to monitor real-time cellular activities. Our device and methodology allowed us to provide a new approach to investigate heterogeneity in drug-induced apoptosis in cancer cells and STAT-1 signaling dynamics in individual cells that established the versatility of our design. With our results we validated the functionality of the chip for multi-dimensional investigations pertaining to signal sensing and processing for a robust response by single cells.

## Results and discussion

### Two-layer microfluidic design for automated delivery of pulsatile stimuli patterns, on-chip

**Fig. 2:**
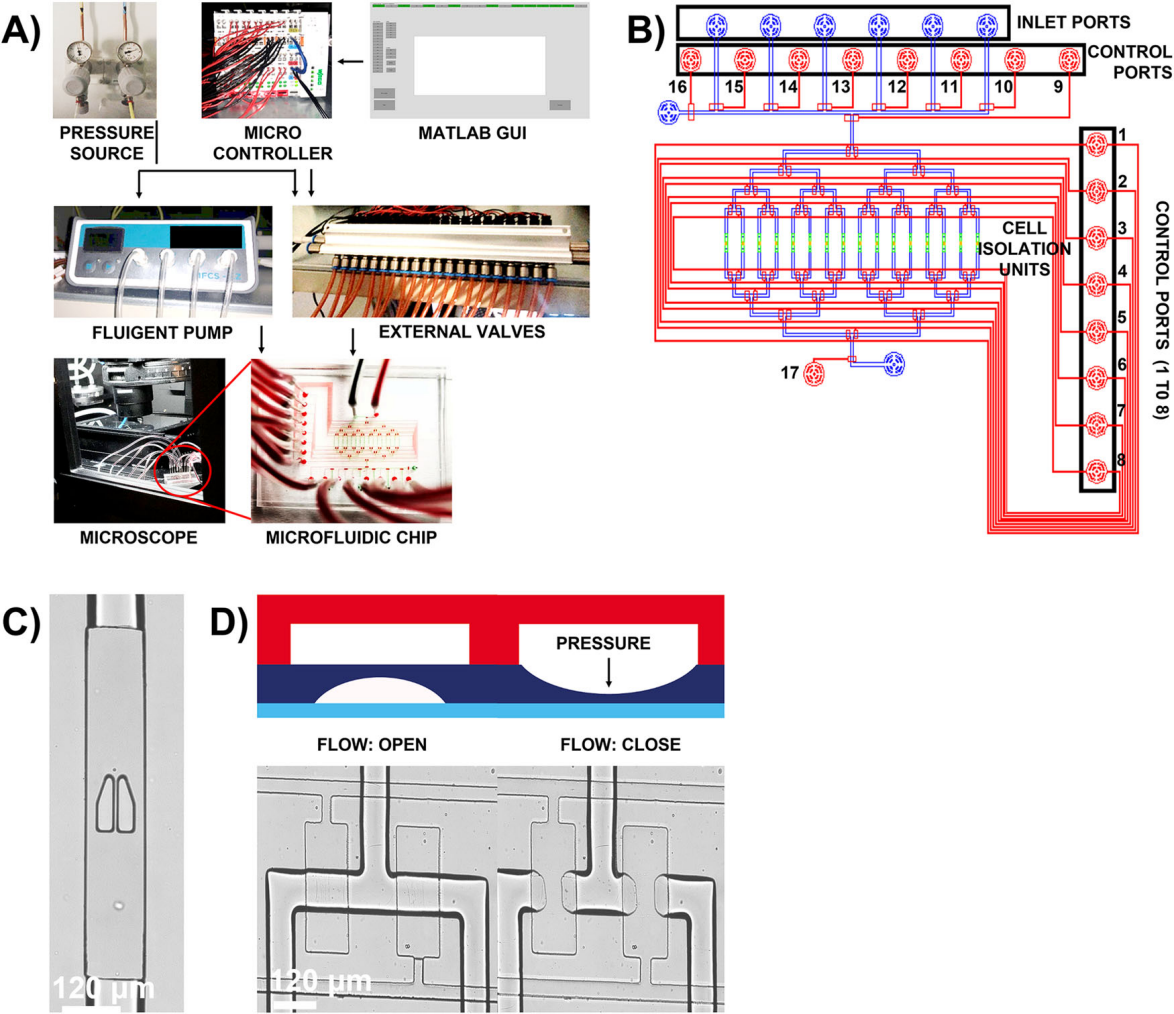
Complete setup of the microfluidic chip, for automation, and detailed description of the chip design. **A** The complete setup of two-layer microfluidic chip comprises of external pneumatic membrane valves and pressure source, to actuate on-chip pneumatic membrane valves open or close, and a graphical user interface, written in MATLAB, to provide instructions and aid in automation of experimental workflow. The microfluidic chip is placed in a stage-top incubator and the cells, on-chip, are imaged using fluorescence microscope. **B** Two-layer microfluidic chip design where flow in sixteen channels (blue) is controlled using eight control lines (red). The inlet channels on the chip are controlled by additional control lines (labeled 9–16) and the outlet is controlled by control port 17. Each flow channel is integrated with cell isolation unit, in green, that has pillar-like traps to physically isolate single cells upon contact. **C** The pillars, fabricated from PDMS, are separated by a small gap of 4 µm and incorporates a V-shaped cup to efficiently hold individual cells in place. **D** The control lines, in the top layer, upon orthogonally intersecting the flow lines, in the bottom layer creates several thin pneumatic push-down membrane valves on-chip. The pneumatic membrane valves are actuated open or close through an external pressure source that control the flow of reagents in the channels.

Our microfluidic chip design (Fig. 2B) is a derivative of mLSI technology and was inspired from the previous designs^19,32,33^. With the motivation to ease the widespread implementation of complex mLSI designs, we derived a simplified version of complex mLSI designs. The top layer of this design comprised of control channels (red) and the bottom layer comprised of flow channels, 19 µm in height (blue) with cell isolation units, height 25 µm. The cell isolation units were designed to integrate PDMS pillars, 25 µm in height, separated by a gap of 4 µm (Fig. 2C) that allowed the physical hydrodynamic cell trapping principle to isolate and trap single cells in each channel when cell suspension was flushed through the channels. In this design, we made 16 microfluidic channels to provide 16 cell isolation units for analysis of individual cells in parallel and at high-temporal resolution.

The orthogonal intersection of control and flow lines resulted in the formation of several pneumatic, PDMS-based, push-down membrane valves (Fig. 2D). The actuation of these membrane valves is regulated with an external pressure source that results in the opening and closing of the flow channels to allow and limit the flow of reagents, respectively. In this design, we organized the membrane valves to form an array that is programmable using binary tree-multiplexing logic. Binary tree-multiplexing logic allowed us to address 2^*N*^ flow channels individually where *N* implies the total number of nodes^34^. At each node, the flow channel bifurcates in two and by implementing a selection process where only one flow channel at each bifurcation is open, 2^*N*^ flow channels are addressable, one at a time. Since our design comprised of 16 cell isolation units, there are in total 4 nodes that allow us to address 16 cell isolation units using a binary logic of 8 digits representing 8 control lines. With a graphical user interface, in MATLAB, we programmed the automatic actuation of the membrane valves, following the multiplexing logic. The graphical user interface communicated the instructions for the actuation of valves via a microcontroller, an external pneumatic valve array, and a pressure source. With the 8-digit logic for each channel (Supplementary Table 1) we addressed each cell isolation unit individually as a function of time to deliver time- and dose-modulated stimuli to single cells.

Multi-layer microfluidic chip designs have contributed vastly towards the understanding of several biological and chemical processes by automating experimental processes^35^. Although the mSLI with trap design was reported previously^19^, the flow mechanism and the efficiency for single-cell trapping remains poorly understood. Thus, we demonstrate in the following sections as to how our design offers better single-cell trapping efficiency, through both simulation and experimental data, that provided us with the needed outcomes. By making these adaptions, we did attain similar levels of control and deliver the needed experimental and environmental conditions for two different applications, making this device a versatile design to be used for future applications.

Our microfluidic device was designed with the motivation to understand temporal dynamics of cellular responses in single cells that result from complex stimulation patterns sensed by cells in their microenvironment. We used our design to highlight the importance of pulsatile stimulation and sensing on downstream cellular responses in two distinct studies using, both, suspension cells, K562, and adherent cells, NIH3T3. The automated and controlled delivery of stimuli to cells was regulated by binary tree-multiplexing logic that also helped us to resolve the problem of cross-contamination and dead volumes^34^. Using this design, we were able to understand how signal fluctuation in the cellular microenvironment influences the activities within apoptotic signaling pathways and interferon signaling pathways.

### Hydrodynamic physical trapping mechanism for efficient trapping of single cells, on-chip

Trapping of single cells in microfluidic channels can be achieved using several methods such as acoustics and optics^36–38^. While all methods are efficiently executable, most of them require the assistance of external equipment to control the process. To overcome this limitation, we exploited hydrodynamic trapping that uses physical barriers to isolate and retain cells at the site of trapping for experimental purposes^39^. This method can further be categorized into contact-based approach, where the cells are required to physically come in contact with pillars or constricted channels to be trapped^19,40^. In our device we used the same principle and integrated the microfluidic channels with PDMS pillars that have V-shaped cup-like structure on top to hold single cells in place (Fig. 2C). Once a singlecell is trapped, the resistance in the middle of the channel increases, thereby, guiding the flow and cells around the pillars for highly efficient deterministic trapping of single cells in the channels.

**Fig. 3:**
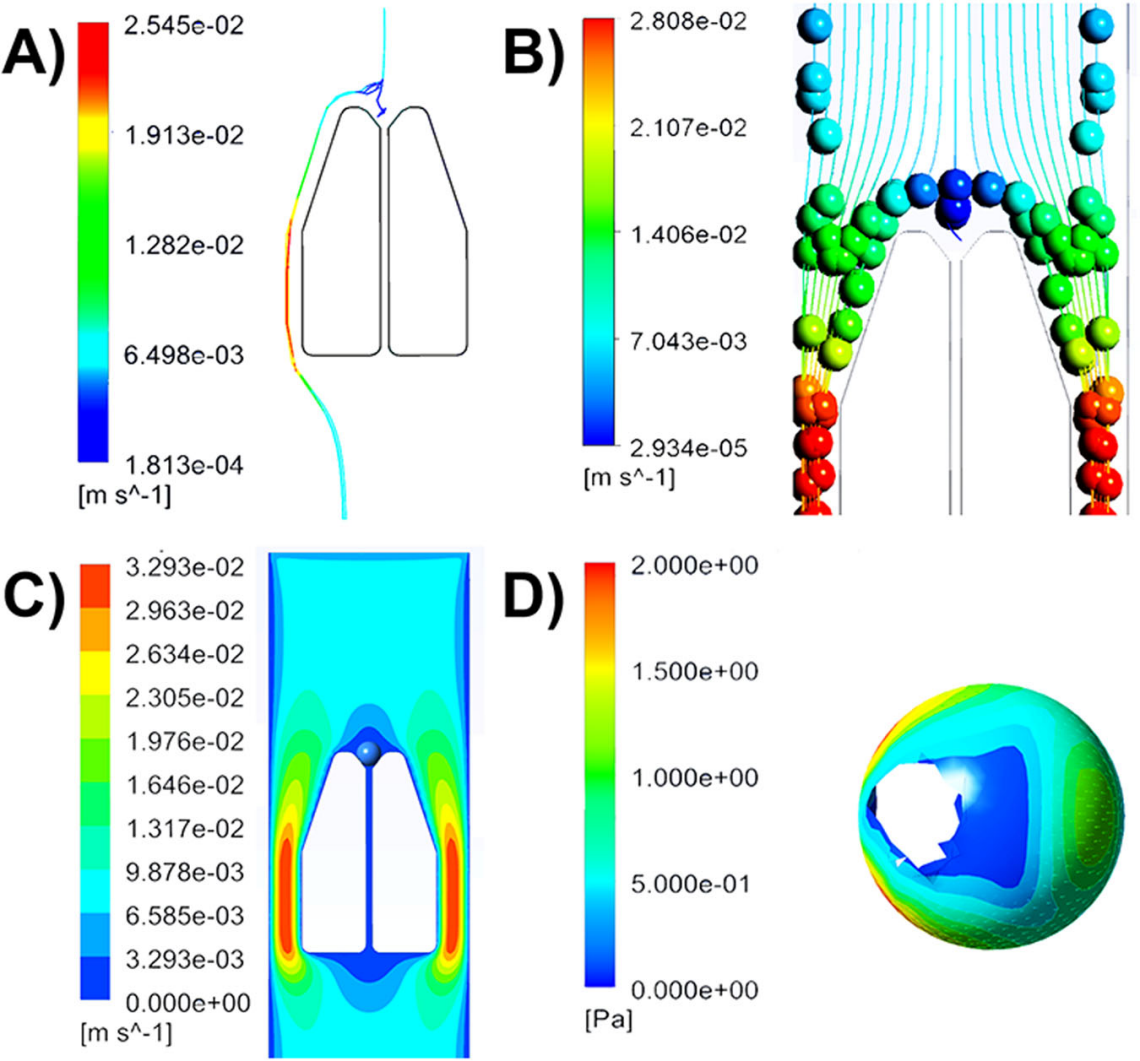
Single-cell trapping mechanism and flow parameters using simulation model. In this design, each of the sixteen microfluidic channels is integrated with pillar-like traps for trapping of single cells on microfluidic chip. **A**, **B** Regardless of whether single cells (**A**) or multiple cells (**B**) are injected at the entrance of the microfluidic channel, simulation model in ANSYS Fluent demonstrated that the cells need to be at the center of the channel to physically interact with the pillars and be isolated. **C** Flow profile in the channel, when a particle of 15 µm is trapped by the pillars. From the flow profile it can be predicted that when other cells enter the channel, they are diverted to the sides of the pillar by virtue of flow profile as there is almost little to no flow through the 4 µm gap between the pillars. **D** Once a cell or a particle has been physically trapped by the PDMS pillars, they will be subjected to shear stress, ranging between 0.25 Pa and 2 Pa, from the flow in the channels. (The white space in the particle, that can be seen as hole, is the part of the section that attaches with the trap during assembly. Upon hiding the traps, that specific section in the rigid particles also disappears giving the impression of hole. This section is not affected by the flow, according to the simulation software.) The particle dimension in this simulation model is 15 µm and the flow rate is 1 µL/min.

We used a simulation model in ANSYS Fluent to visualize and validate our approach of hydrodynamic cell trapping using a particle size of 15 µm. We modeled this size with respect to K562 cells and NIH3T3 cells used in this study, which have average diameters of 17 µm and 15 µm, respectively. In the first simulation model, a single rigid spherical particle of 15 µm was released in the middle of the channel at a flow rate of 1 µL/min (Fig. 3A). The results demonstrated that the particle followed the central flow line and encountered the pillars. If we released additional particles after a time gap, these particles were diverted to the sides of the pillar. In the second model (Fig. 3B), multiple particles of 15 µm were released randomly in the channel at the same flow rate to demonstrate that almost none of the particles, except the ones that are in the middle of the channel, physically interact with the pillars to get trapped. Both simulation models demonstrated that particles in the middle of the channel will be efficiently trapped by pillars whereas every additional particle will flow around the side. This pattern can be attributed to the pillar design that has slanting edges at the top causing most flow in the channel and particles to divert rather than converge to the center. The flow profile in the channel, upon trapping a particle, was also investigated in this study (Fig. 3C) and validated our hypothesis. We created an assembly with a rigid 15 µm particle at the pillar and added flow in the channel at 1 µL/min to demonstrate that the flow around the trapped particle is almost negligible due to high resistance. While we used K562 cells and NIH3T3 cells in our study, cells with a diameter ranging between 10 µm and 18 µm can also be trapped using this design as the flow follows similar patterns in the channels regardless of the particle size (Supplementary Fig. 2).

With the flow simulations, we also measured the wall shear stress (Fig. 3D) at 1 µL/min and obtained a maximum value of 2 Pa at the surface of the particle. Previous studies have measured elastic properties of cells and some studies also suggested that cells have viscoelastic behavior^41^. This establishes that under certain stress profiles, cells will undergo deformation by virtue of its Young’s modulus^41^. By modeling the cells as rigid particles, owing to the limitations of the software, we could not measure the stress-induced deformation on cells. Understanding shear stress is important to ensure that the cells are not exposed to shear stresses that are far higher than those experienced in physiological conditions, as this could influence the morphology of cells or induce stress-mediated responses. Whilst there is a wide body of research on the effects of shear stress on other cell types, the effects on immune cells have not been fully explored. However, research has shown that shear stress in human arteries range between 1 Pa and 6 Pa, whereas veins range between 0.1 Pa and 1 Pa resulting from flow rate in the blood vessels^42^. Although our simulation model shows that the cells will experience shear stress in the higher range, this high value is only across a very small area whereas most of the surface experiences low range of stress. During the course of the experiment, cells will move to the bottom of the channel under the influence of gravity or adherent cells will start to stretch over the surface and will experience different values of shear stress in the channels (Supplementary Fig. 3). We also investigated the shear stress experienced by particles of sizes 10 µm and 18 µm, to ensure that these values are in the nominal range when subjected to flow rate of 1 µL/min (Supplementary Fig. 4). Using our simulation model, we have validated the efficient cell trapping principle of our microfluidic design and have successfully implied that influences from flow will not influence cellular behavior, which is often a concern when using microfluidic designs for biological applications.

**Fig. 4:**
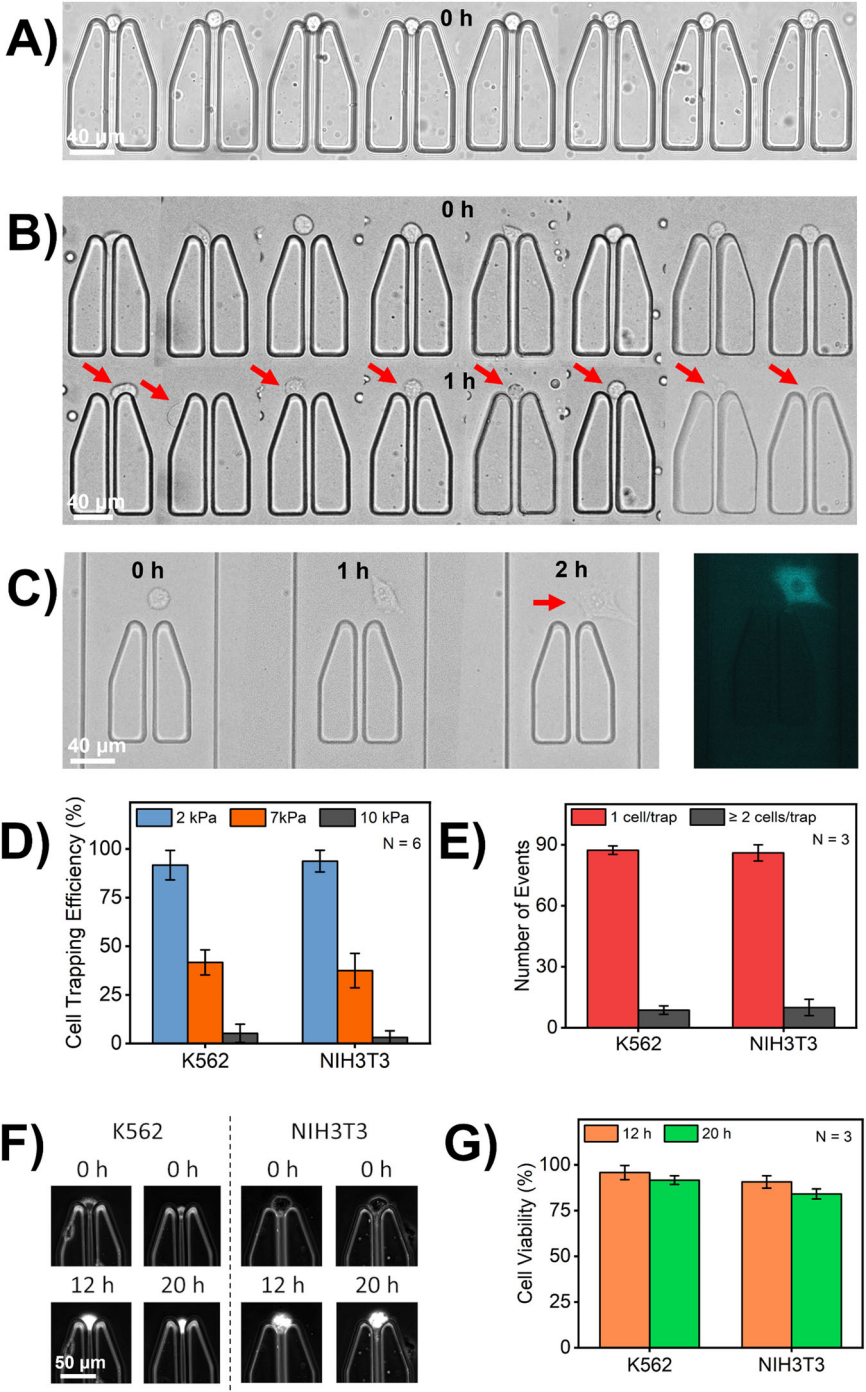
Efficient cell trapping demonstrated by the microfluidic chip. **A** This design has demonstrated high efficiency in trapping suspension K562 cells that can be treated with stimuli to monitor cellular response immediately after seeding in the channels. **B** This design is also compatible with adherent NIH3T3 cells. Fibroblast cells, when flushed in the channels are round and over the time will sink to the bottom of the channel to stretch which is promoted with fibronectin coating. Additionally, these cells also migrate in and out of the trap and within the channel. **C** A single fibroblast, that took 1 h, to stretch in the channel and after additional 1 h stretched completely for distinct visualization of the cytoplasm and nucleus. The blue-fluorescent signal is from CFP-labeled IRF7 transcription factor that resides in the cytoplasm until activation. **D** Highly efficient and consistent trapping of single cells in our device with over 90% channels being able to isolate cells, both adherent and suspension, at low pressures (*N*: Number of individual events). At higher pressures, of around 10 kPa, the cells squeezed through the middle of the PDMS pillars and were difficult to retain. Error bar represents mean ± SD. **E** The cell trapping experiment was repeated multiple times on three different microfluidic chips to determine the design’s reusability and reproducibility. For each individual chip, we observed more than 85 events with single-cell trapping and very few events with more than one cell in channel. Error bar represents mean ± SD. **F** Representative microscopic images of viable K562 cells NIH3T3 cells that were cultured in microfluidic channels. **G** K562 cells and NIH3T3 cells, cultured on the chip, showed high viability of over about 80% through media exchange. Error bar represents mean ± SD.

We flushed the cells in the channels by actuating each cell isolation unit individually. The cell trapping efficiency and mechanism on the chip resembled the first simulation model where individual cells in the middle of the channel would enter the cell isolation unit at short time intervals and were trapped at the pillars. We used, both, suspension K562 cells (Fig. 4A) and adherent NIH3T3 cells (Fig. 4B, C) to highlight that we can trap different kinds of single cells in all the channels under optimal conditions of input pressure and cell seeding concentration (materials and methods). Our cell trapping efficiency was consistently high (over 90%), for both K562 and NIH3T3 cells (Fig. 4D). While we observed very minor events of more than one cell in each unit, most of the channels indeed trapped and isolated single cells only (Fig. 4E). With the cell trapping efficiency, input pressure plays a crucial role as with high input pressures, cells easily squeezed through the 4 µm gap between the pillars and were flushed away. As shown in Fig. 4D, our cell trapping efficiency reduced with increased input pressure. Input pressure higher than 2 kPa proportionally increased the flow rates in the channels causing cells to either diverge or squeeze through the gap. Input pressures ranging between 1 kPa and 2 kPa ensured that more cells stayed in the center as demonstrated in the first simulation model. Similar pressure ranges were maintained during the exchange of media and stimuli in the channels to minimize cellular deformation resulting from flow in the channels. In addition to input pressure for seeding cells, the number of cells per mL in cell suspensions is also critical. Upon trapping the K562 cells in the channels, they can instantly be cultured by exchange of media. Alternatively, the cells can also be treated with stimuli directly after being trapped. When flushing fibroblasts in the channels, it is essential to take into consideration the cell seeding density as a very high cell concentration can cause the cells to adhere to one another resulting in isolation of more than one cell. Also, fibroblast cells have the tendency to migrate and stretch either on the surface or around the pillars over time (Fig. 4B, C) and must be given adequate time to adhere before reagent exchange. We also used human peripheral blood mononuclear cells (PBMCs) to estimate the trapping efficiency of device with primary cells, which are also smaller in size and have higher tendency to stick. While our device was compatible with primary cells as well, we observed lower trapping efficiency in comparison to the cell lines used (Supplementary Fig. 5, Supplementary Fig. 6). This can be attributed to the irregular and smaller cell size of these cells that can squeeze through the 4 µm gap between the pillars. For future research with primary cells, the trap design requires modifications to match the size range of small primary cells (e.g., T cells) and the implementation of modified cell loading techniques to circumvent cellular adhesion and loss of cells. We also observed that over 75% of both cell types retained in the channels for 6 h of which NIH3T3 cells had a higher retention rate given their adherent nature (Supplementary Fig. 7). Following the retention rate, it was also noteworthy that, both, K562 cells and NIH3T3 cells demonstrated high viability (measured separately in independent experiments), with a medium exchange, with over 80% of the cells being viable over a period of 20 h, making the design suitable for long-term experiments (Fig. 4F, G).

Physical hydrodynamic trapping in various forms such as arrays and micro trenches has been widely implemented in the past research^43–45^. These designs offer high-throughput solution to single-cell investigation and have provided reliable results for various, distinct biological research applications. With our design, we have greater control with cell trapping as we address each unit individually that allowed us to ensure that each unit confines a single cell. Furthermore, these trapping units provide an isolated environment for single cells, to guarantee results at single-cell level^46,47^. This cannot be achieved in either arrays or micro trenches, since secreted molecules can diffuse between single cells to establish intercellular communication. Our results have demonstrated the efficient trapping, retention, and viability of cells in microfluidic channels, which is important while performing single-cell investigations.

### Apoptosis in K562 cells, at single-cell level, shows varied response patterns

Apoptotic signaling pathways hold significance to cell biologists as it regulates programmed cell death resulting from either intrinsic factors initiated by mitochondria, or extrinsic factors upon activation of surface receptors^48^. The downstream signal transduction of this pathway releases caspase 3 protein for degradation of genetic materials and proteins with high control^49^. Understanding the underlying dynamics and mechanism of apoptosis is of special interest to researchers as cancer cells mutate to bypass the cell destruction process and can proliferate endlessly, thereby proving to be a roadblock in cancer cell therapies^50^. However, in a tumor microenvironment, not all cells bypass the cell destruction process. Rather a fraction out of multiple cells that is not easily predictable given that this phenomenon occurs in individual cells^51^. This highlights the relevance of single-cell investigation of apoptosis induction in cancer cell lines to further explore the interplay of factors that influence this phenomenon. Hypothetically, cells could have heterogeneous responses in caspase-mediated apoptotic pathways. This variance in cellular behavior may be further influenced by the cell’s exposure to stimuli to a great extent. Henceforth, in this study, we investigated the induction of apoptosis of K562 cells when treated with DMSO. DMSO has widespread biomedical applications as it is a popular solvent for many medical compounds and is also used for cryopreservation of cells in addition to several other applications^52^. Even though the use of low concentrations of DMSO is approved for several medical applications worldwide, given its ability to penetrate cell membrane, a higher concentration of DMSO has demonstrated toxic effects on cells^53^. While the effect of DMSO has been variable on different cell types, doses higher than 1% have indicated to have negative effects on cells, which is also dependent on the time duration of exposure. Therefore, we believe that it is a good candidate to study temporal dynamics of apoptosis induction in single K562 cells using our device^54^.

**Fig. 5:**
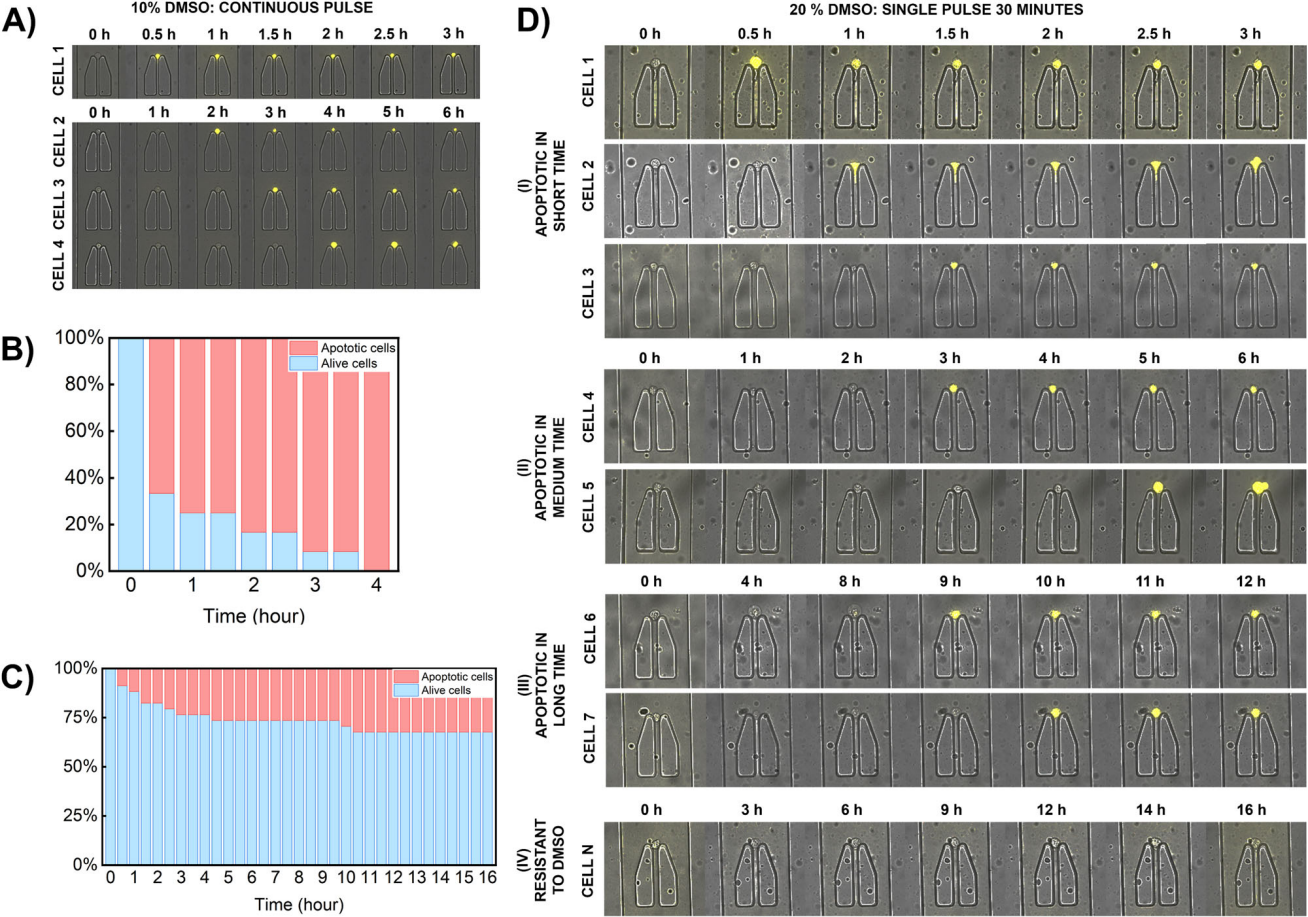
Results from single-cell apoptosis study performed on this microfluidic chip. **A** Time-lapse microscopic images of apoptosis In K562 cells when treated continuously with 10% DMSO concentration in culture media. During apoptosis the cells emit fluorescence from the binding of caspase dye to DNA, after the release of proapoptotic proteins. **B** In this condition (presented in **A**), 100% cells died over a course of 4 h, with, over 75% of cells being apoptotic within the first one hour. **C** When cells were treated with 20% DMSO solution in culture media for 30 min, apoptosis was observed in only a fraction of cells, around 33%. A large fraction of cells were non-apoptotic until 16 h. Under this condition high variation in response time was observed resulting from delivering the stimuli to individual cells in pulses rather than continuously. **D** Time-lapse microscopic images of K562 cells (referring to results in **C**) when treated with single pulse of high concentration, 20% DMSO solution. The response time in each individual cell varied over the time of 16 h as seen in this representative image.

We treated individual K562 cells with different concentrations of DMSO for different time durations to observe a variation in response time in single cells. The apoptosis in cells was monitored by the addition of caspase-3/7 dye, which was added to the cell suspension before seeding the cells. This helped us to rule out the number of cells that died before or during cell seeding (Supplementary Fig. 8). When using 10% DMSO concentration in the culture media continuously, most cells exhibited a fast response to the stimuli with 100% of the cells undergoing apoptosis within the first four hours (Fig. 5A, B). However, when observing this response in individual cells, each cell had a different timeline of apoptosis. While approximately 75% cells were apoptotic within the first one hour, a relatively smaller fraction of cells, 25%, showed a delayed response. Additionally, we also treated individual cells with a higher concentration of DMSO, 20%, in RPMI for thirty minutes (Fig. 5C, D). The variation in timeline of apoptosis induction with this condition was much higher. The cells were continuously monitored for 16 h during which time only 33% cells showed apoptotic response (excluding cells that were found dead initially). Even within this subpopulation, cells had varied timeline with approximately 24% of the analyzed cells responding faster within 3 h compared to other cells, approximately additional 9%, that responded after 3 h to as late as around 10 h. Also, this response was observed until the tenth hour after which the remaining cells were viable in the microfluidic channels.

Our results demonstrated that distinct exposure pattern of apoptosis-causing reagents will impact the cells differently. Previous research has also presented an array platform that allows the screening of anti-cancer drug on microfluidic-chip, however with this design we cannot nullify intercellular communication between cells^55^. With our design, we observed different subgroups that responded differently to DMSO to present results that were at absolute single-cell resolution and not cumulative. In this work, the only extrinsic stimuli resulting in these distinct responses was DMSO. We wanted to use this experimental model to highlight the significance of pulsatile stimulation on assessing cellular behavior with the perception that at the level of a single cell, there is a variation in response. Variations in levels of death receptors, mitochondrial levels, and node proteins i.e. caspase can result in this variability^56,57^. While we tested the effect of two different concentrations of DMSO for two different exposure times, it is also possible to further stimulate the live cells to study whether additional exposure is required to kill K562 cells or they are inherently resistant to a given chemical. Given the ability to wash the cells and exchange reagents or media, this experimental model validates the relevance of this design for implementing drug-screening assays.

### Distinct temporal activation of STAT-1 at single-cell level

The activity of transcription factors is regulated by biochemical signals that cells sense in their microenvironment, which has shown variability at single cell level^58^. The observed variations in response can be attributed to multiple factors including ligand simulation that is delivered or sensed in pulses^59,60^. At the level of a single cell, signaling pathways and corresponding transcription factor activities can either respond to a pulse of stimuli or not and sometimes require more than one pulse to respond^61^. This response dynamics is not just dependent on the time duration of exposure to stimuli but concentration and the rest time between the pulses also play an important role in this process^61^. By using pulsatile stimulation at single-cell level and microfluidic platforms, researchers have obtained mechanistic insights into deterministic processes that can be exploited towards fine-tuning cellular fates^62^. Inspired by previous research on NF-κB, we here probed STAT-1 dynamics using a similar approach^62^. The binding of IFNγ to the interferon-gamma receptor (IFNGR) activates STAT-1 protein to form a homodimer that translocates to the nucleus to bind with gamma-activated sequence (GAS) leading to transcription of interferon stimulatory genes (ISGs)^63^. Similar to other signaling pathways, we hypothesized the influence of time and dose-modulated delivery of IFNγ on STAT-1 protein, which we investigated using our microfluidic platform.

**Fig. 6:**
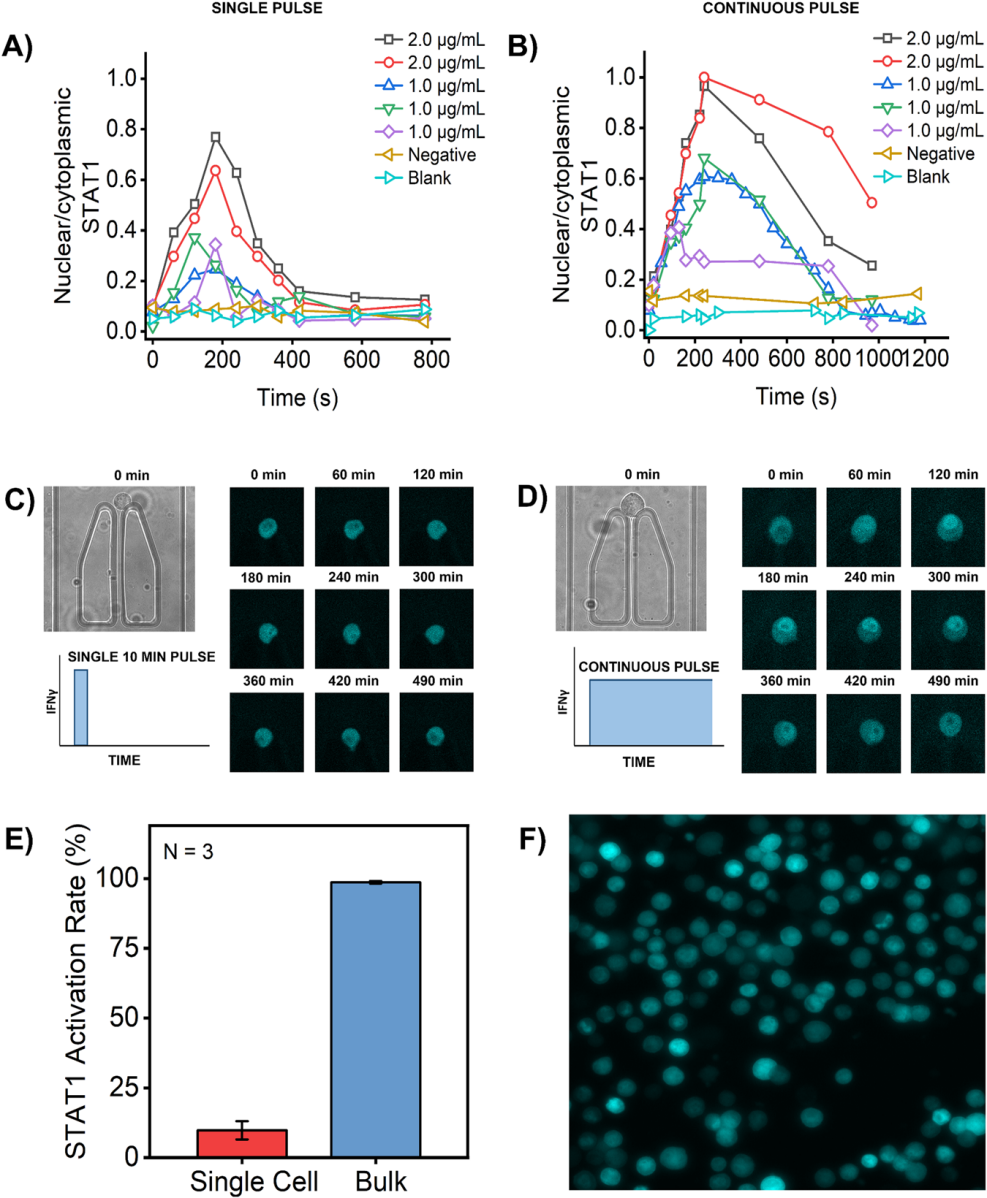
Differential STAT protein activation profile upon stimulation with IFNγ. **A** Translocation was measured by plotting the ratio of fluorescence intensities between the nucleus and the cytoplasm. At different concentrations of 1 and 2 µg/mL distinct translocation patterns, with respect to time, were observed when cells were treated with a single 10 min pulse of IFNγ. **B** At concentrations of 1 and 2 µg/mL distinct translocation patterns, with respect to time, were observed when cells were continuously exposed to IFNγ which was also different from the patterns observed due to short single pulse exposure. **C**, **D** Representative images of NIH3T3 cells that showed STAT-1 activity when treated with 10 min pulse (left) and continuous exposure (right) of IFNγ. **E** The bar graph displays the percentage of cells demonstrating STAT-1 activation in single cells in microfluidic channel, which was much lower compared to percentage of activated cells in bulk. *N* = 3. Error bar represents mean ± SD. **F** Representative image of cells in population study that showed higher percentage of active cells in comparison to single-cell study.

To understand the variation in the activation and response dynamics of STAT-1, we stimulated single NIH3T3 fibroblasts, trapped and isolated in microfluidic channels, in two different ways. In the first set of experiments, we stimulated the cells with one pulse of IFNγ for ten minutes and in the second set of experiments the single cells were treated continuously with IFNγ, i.e. the channels were flushed continuously with IFNγ. Interestingly, our results showed distinct STAT-1 response dynamics for the two different stimulation modes (Fig. 6A, B). This can also be visualized in representative microscopic images presented in Fig. 6C, D. When cells were stimulated for only ten minutes, the activation of STAT-1 was steep with fast translocation to nucleus and back in comparison to the continuous stimulation wherein we observed longer residence of STAT-1 in the nucleus. Additionally, varying the concentration of IFNγ, while keeping the exposure time consistent, resulted in a variable response. We observed that stimulation with a higher dose resulted in stronger amplitude signals, showing a dose–responsive curve of activated single cells. Furthermore, we also noticed cells that did not respond to IFNγ stimulation, which we concluded by making a comparison between our negative control (cells not treated with the stimuli) (Supplementary Fig. 9). The results from our single-cell study, drove our curiosity to investigate whether the fraction of cells responding to the stimuli in bulk follow a similar order. This was, however, not the case as demonstrated in Fig. 6E, F. Comparing the fraction of responding cells, when stimulated continuously, revealed a 9-fold increase in this population in our bulk studies.

Similar to other transcription factor activities, STAT-1 activity is also a tightly regulated process, governed by several factors including the stimuli. The distinct patterns observed upon varying the concentration of IFNγ and mode of exposure is indicative of the fact that STAT-1 activity is regulated by stimuli that influenced cellular decisions observed in this study. At higher concentrations of stimuli, regardless of the stimulation pattern, the response was stronger in comparison to the low concentrations to imply dose-dependent activation of STAT-1 signaling protein. Differences in dynamic curves of STAT-1 also imply time dependency of STAT-1 activity. In addition to the responding cells, we also observed non-responding cells that are of equal significance as they did not meet the activation threshold to result in STAT-1 activity. Whether they require an additional pulse of stimulation or higher concentration of stimuli, is of interest to explore in future investigations. Furthermore, the cells showed greater STAT-1 activity at the population level than at the single-cell level. The difference between single-cell activity and bulk activities could be attributed to cellular communication, such as paracrine signaling, which is negated in our single-cell approach^10^. Previous research also showed limited immune response in single cells as compared to cells activated in bulk^10^. The transcription of ISGs, from STAT1 activity, results in several mediators, i.e., proteins and cytokines, that will be communicated to neighboring cells to establish robust immunity^64^.

Pulsatile stimulation in combination with single-cell investigation is a powerful methodology that has allowed researchers to distinguish between deterministic and stochastic cellular decision-making process^65,66^. Studies have demonstrated that varying levels of receptors, kinases or gene states drive cell-to-cell variability and result in distinct cell fates^61^. While in this study, we did not quantify the volumes of receptor or node proteins, the variability observed in our results most likely originates from varying levels of proteins in each individual cell. Furthermore, mathematical models have revealed that a high Hill coefficient reaction further amplifies the different response dynamics in signaling pathways^61^. In addition to aforementioned factors, the effect of concentration and time modulation of stimuli on STAT-1 in individual cells was also evident in our study to imply that modulation of stimuli can tune immune responses. Given that cells demonstrated stronger response at higher concentration and vice vera is suggestive of analogue encoding of IFNγ dose information by STAT-1 dynamics. Furthermore, with different exposure patterns, we have shown the regulation of downstream responses. With a short pulse of 10 min, the residence in the nucleus was also short, giving cells enough time for recovery to respond to the second pulse if required. However, when subjected to continuous stimulation, STAT-1 showed longer activity in response to the long exposure time. Further, the response dynamic curve was consistent for all concentrations for each given stimulation pattern. So, by recognition of consistency in response patterns we concluded underlying heterogeneity in STAT-1 signaling dynamics, a property that cellular systems utilize to filter noise and to ensure robustness of the system.

## Conclusion

Biological systems and cell types are often classified based on their functional response to the stimuli sensed by the cells from their microenvironment. Cells, equipped with different receptors, recognize and process these biochemical cues to orchestrate response generation. These biochemical cues are often released and sensed in patterns such as pulsatile, ramp-up, ramp-down, and sinusoidal to directly influence downstream signal processing mechanism^5^. For accurate understanding of signal processing and cellular responses, it is, therefore, important to integrate new methodologies that allow for precise delivery of stimuli, as found in vivo. Additionally, single-cell investigation using pulsatile stimulation adds value to research as it aids in the recognition of functionally heterogenous cell populations whose response deviates from normal behavior in absence of paracrine communication from neighboring cells. It also helps to differentiate whether certain response patterns are intrinsic in nature or are influenced by environmental cues. To facilitate studies involving such multi-dimensional questions, we presented a two-layer microfluidic device that delivers distinct patterns of stimuli to isolated single cells, trapped using hydrodynamic mechanism, in microfluidic channels. Furthermore, the integration of multiplexing logic, for automation of the experimental workflow on this chip, allowed us to provide stimuli to individually cultured adherent and suspension cells in pulsatile patterns. While the current version of this design has 16 cell isolation units, with design modifications and additional channels, there is a possibility to increase the throughput of this device accordingly.

To benchmark our platform and translate interesting research lines on microfluidic chips, we studied apoptosis in K562 cancer cell line and observed that individual cells had different timelines of apoptosis in response to DMSO. This channels the hypothesis that apoptotic pathways in cancer cells, when treated with different reagents, can have underlying heterogeneity and the effect of reagents must be assessed at single-cell level to determine the right dosage and type. Additionally, we also monitored the activation of STAT-1 in NIH3T3 cells in response to IFNγ, delivered to individual cells in pulse and continuously. STAT-1 in individual cells responded differently to different stimuli pattern that ranged between sharp, gradual, and no response. This is the first time that both, apoptosis dynamics and STAT1 response dynamics at single-cell level have been studied on a mLSI platform to provide new methodology of investigations. With both the studies we were able to emphasize the relevance of pulsatile signal delivery and single-cell investigation in understanding intracellular signaling pathways. While we highlighted the relevance of our two-layer microfluidic chip design with two distinct applications, our device can also be used for several other research lines to comprehend cellular responses with greater depth.

## Methods

### Microfluidic-chip design

The two-layer microfluidic chip was designed in AutoCAD, 2017 (Autodesk, USA).

### Silicon wafer (master mold) fabrication

To fabricate two-layer microfluidic polydimethylsiloxane (PDMS) devices, two distinct silicon wafers with fabricated channels were required (Supplementary Fig. 1). The 2D model of the design, created in AutoCAD, was used to print the photomasks for photolithography. For the control channels and flow channels, negative and positive polarity foil masks, respectively, were ordered from CAD/ART Services, Inc. (USA), whereas for the cell isolation units, negative polarity chrome mask was ordered from JD Photo Data (UK). For this design, the control channels were fabricated using SU-8 3025 negative resist (Microresist Technology GmbH, Germany) to obtain rectangular cross-section of height 50 µm. The resist was spin-coated at a speed of 1400 rpm and exposed to UV light with an energy of 250 mJ/cm^2^. The fabrication of flow channels and cell isolation units in the flow layer was completed in two steps. First, the flow channels were fabricated by spin-coating AZ 9260 (Microchemicals GmbH, Germany) positive resist on a hexamethyldisilazane hexamethyldisilazide (HMDS) primed wafer at a spin speed of 900 rpm to obtain rectangular cross-section of height 16 µm. The positive resist was exposed to UV light with an energy of 960 mJ/cm^2^. To round the channels, the fabricated wafer was then placed in an oven at room temperature and heated to a temperature of 160 °C overnight. The reflowed wafer was then spin-coated with SU-8 3025 negative resist (Microresist Technology GmbH, Germany) at a spin speed of 3000 rpm to obtain a rectangular cross-section of height 25 µm for the cell isolation units and exposed to UV light with an energy of 167 mJ/cm^2^. The prepared wafers were salinized using chlorotrimethylsilane (TMCS) (Sigma Aldrich, The Netherlands) to ensure that cured PDMS can easily be peeled off from the wafers.

### PDMS microfluidic device fabrication

For fabrication of two-layer microfluidic chip, two PDMS layers, flow and control, were cured separately. The flow layer was prepared by spin-coating a mixture of PDMS base and curing agent in ratio 20:1 (20 g to 1 g) and the thicker control layer was prepared by pouring a mixture of PDMS base and curing agent in ratio 5:1 (35 g to 7 g) on respective silicon wafers. Both layers were cured separately at 80 °C for 20 min. Once cured, the control layer was peeled from the silicon wafer, holes were punched to open the control ports, and the control layer was aligned carefully on top of the flow layer that was then placed in an oven, set at 80 °C, to thermally bond the two layers. After thermal bonding the two layers, the complete device was removed from the wafer, holes were punched to open the flow channels, and the flow channels were closed by bonding the device to a glass slide using plasma. The plasma was then deactivated by keeping the completed microfluidic chip in oven, set at 80 °C, overnight and the device was ready to be used the following day.

### Microfluidic-chip setup and automation

The operation of the microfluidic chip was fully automated and controlled using a script that was written in MATLAB (Mathworks, USA). This microfluidic chip was operated using normally open external pneumatic valves (FESTO, Germany) connected to a computer, via a microcontroller, and a pressure source. The control ports on the microfluidic chip were connected to their corresponding units on the external pneumatic valves via flexible tubing. If the value fed to the external pneumatic valves was 0, then the external pressure line opened that in turn pressurized the membrane valves of the corresponding control lines, on-chip, to close the channels and vice-versa. For this microfluidic chip, 17 units of external pneumatic Festo valves were required for complete automation of the experimental workflow, which was read and executed from an EXCEL sheet prepared in the same folder as the MATLAB files. The overall setup of our system is presented in Fig. 2A.

### Simulation model in ANSYS fluent

The microfluidic chip was designed in Autodesk AutoCAD 2017. Additionally, SolidWorks (USA) was used to create three-dimensional geometry of the cell isolation unit that was imported to ANSYS (USA) Fluent module for simulation. In addition to the channel, rigid spherical particles of different sizes, as a representation of different cell types, were also designed in SolidWorks. Multiple assemblies were created with a particle at the center to represent single cells in microfluidic channel upon isolation. Different models and assemblies were used to investigate the flow profile and shear stress on the particle using steady-state model, and hydrodynamic trapping mechanism using transient solver. In the transient solver, macroscopic particle model (MPM) was loaded that allowed for the simulation of particles that can interact with geometrical structures under flow conditions. The particles were assigned with different physical properties, such as size, density, and the coefficient of restitution (CoR), a measurement of how elastic the collision is between two objects. Once all the properties were defined including boundary conditions, the simulation model was started.

### Cell harvesting, seeding, and culture on the microfluidic chip

K562 cells, obtained from ATCC (USA), were cultured according to the protocol suggested by ATCC with minor modifications. The cells were cultured in Roswell Park Memorial Institute (RPMI) (Sigma Aldrich, The Netherlands) medium supplemented with 10% fetal bovine serum (FBS) (Sigma Aldrich, The Netherlands), and 1% penicillin and streptomycin. Murine fibroblast NIH3T3 reporter cell lines, with fluorescently labeled transcription factors^67^, were cultured in standard Dulbecco’s Modified Eagle Medium (DMEM) (Sigma Aldrich, The Netherlands) medium that was supplemented with 10% fetal calf serum (FCS) (Sigma Aldrich, The Netherlands), and 1% penicillin and streptomycin.

To flush cells in the microfluidic chip, cells were taken from culture, spun down, and re-suspended in fresh culture medium to reach a final concentration ranging between 1.0 × 10^6^ cells/mL and 2.5 × 10^6^ cells/mL for K562 cells and ranging between 0.5 × 10^6^ cells/mL and 1.5 × 10^6^ cells/mL for NIH3T3 cells, respectively. For NIH3T3, which are adherent, cells were first treated with trypsin to detach them from flask. Furthermore, the glass surface of the microfluidic channels was also treated with fibronectin (Sigma Aldrich, The Netherlands) at 100 ng/mL to promote cellular adhesion. When using confocal imaging for monitoring cellular response, the adherent cells were imaged as rounded and coating of the glass surface with fibronectin was skipped. The cell suspension was drawn in a tygon tubing (Darwin Microfluidics, France) with the help of a syringe. One end of the tubing was inserted into one of the inlets on the microfluidic chip with the help of connector pins (Darwin Microfluidics, France) and the other end was connected to one of the units on pressure-driven MFCS EZ pumps (Fluigent, Germany). The pressure on the unit was adjusted between 10 mbar and 20 mbar after actuating the respective inlet and outlet on the chip open followed by actuating each cell isolation unit open till a cell is trapped in each unit. Optimal conditions for cell culture were maintained by placing the microfluidic chip in a stage top incubator (Okolab, Italy), set at 5% CO_2_ and 37 °C. Additionally, the cells were also cultured for more than 20 h in the microfluidic channel through the respective medium exchange, and the end point viability of the cells was observed using Calcein AM cell-permeant dye (ThermoFisher, The Netherlands) prepared at a concentration of (2 µg/mL) in culture medium.

### Measurement of apoptosis dynamics in K562 cells

Apoptosis induction in K562 cells was measured by treating the cells with different concentrations of dimethyl sulfoxide (DMSO) (Sigma Aldrich, The Netherlands). The early apoptosis response in the cells was recognized using caspase-3/7 dye (Thermo Fisher, The Netherlands). The chip was prepared as described above and air from the channels was flushed out with RPMI media solution containing caspase dye at a concentration of 1 micromolar (µM), to identify cells that are in the apoptotic stage when trapped. Once the cells were trapped, they were treated with two different concentrations of DMSO. For the first dataset, the cells were treated with 10 % DMSO, in RPMI media with 1 µM caspase dye, by flushing the stimuli continuously over the time course. For the second dataset, the single cells were treated with 20% DMSO, in RPMI media with 1 µM caspase dye, for 30 min after which the stimuli were exchanged with fresh RPMI media containing 1 µM caspase.

### Measurement of STAT-1 translocation dynamics in NIH3T3 cells

The STAT-1 signaling pathway, in single fibroblast cells in microfluidic channels, was activated with interferon γ (IFNγ) (GenScript, The Netherlands) prepared at different concentrations of 1 µg/mL and 2 µg/mL in a standard DMEM culture medium. Prior to seeding the cells, the air was flushed out with a DMEM medium. Once the cells were trapped and isolated in the channels, they were flushed with IFNγ for ten minutes, after which the stimuli were washed away with DMEM culture medium, or for the entire duration of the experiment.

### Image acquisition and processing

Images were acquired using Nikon Eclipse Ti2 (Nikon, Germany) microscope. Depending on the data being acquired, individual cells in each cell isolation unit were imaged using different channels. Apoptotic K562 cells were imaged with yellow fluorescent protein (YFP) channel using fluorescence settings. Translocation dynamics of STAT protein was acquired with cyan fluorescent protein (CFP) channel using either fluorescence settings, for cell culture or confocal settings, for monitoring STAT translocation. The acquired images were analyzed using FIJI/ImageJ software (National Institutes of Health, Bethesda, USA). In order to quantify STAT1 nuclear/cytoplasmic (N/C) fluorescent ratio, we drew the outlines of nucleus and cytoplasm areas for each individual fibroblast, and then measured the nuclear and cytoplasmic fluorescence intensities.

### Statistics and reproducibility

The data, from the obtained images, was processed using Origin (OriginLabs, USA) software. Each experiment was repeated at least thrice and the collective data were represented as mean value with standard deviation.

### Reporting summary

Further information on research design is available in the Nature Research Reporting Summary linked to this article.

### Supplementary information

Tel_PR file

Supplementary Information

Reporting Summary

## Data Availability

The datasets generated during and/or analyzed during the current study are available from the corresponding author on reasonable request.

## Code availability

The codes generated during and/or analyzed during the current study are available from the corresponding author on reasonable request. The code for automation of microfluidic chip was written in MATLAB 2019, which is available from the corresponding author upon request.
