# Supplementary material for: Microfluidic chip for precise trapping of single cells and temporal analysis of signaling dynamics
Source: Commun Eng. 2022 Jul 25;1:18. doi: 10.1038/s44172-022-00019-2 (PMC10955935; doi:10.1038/s44172-022-00019-2)
Supplement: Supplementary file 2 — Supplementary Information [file 44172_2022_19_MOESM2_ESM.pdf]

Supplementary Information

Microfluidic chip for precise trapping of single cells and temporal analysis of signaling dynamics

Nidhi Sinha[1,2 *], Haowen Yang[1,2 *], David Janse[1], Luc Hendriks[1], Ulfert Rand[3], Hansjörg Hauser[3], Mario Köster[3], Frans N. van de Vosse[4], Tom F. A. de Greef[2,5], Jurjen Tel[1,2 #]

Affiliations

1. Laboratory of Immunoengineering, Department of Biomedical Engineering, TU Eindhoven, 5600 MB, Eindhoven, Netherlands

2. Institute of Complex Molecular Systems, TU Eindhoven, 5600 MB, Eindhoven, Netherlands

3. Model Systems for Infection and Immunity, Helmholtz Centre for Infection Research, 38124, Braunschweig, Germany

4. Cardiovascular Biomechanics Group, Department of Biomedical Engineering, TU Eindhoven, 5600 MB, Eindhoven, Netherlands

5. Computational Biology Group, Department of Biomedical Engineering, TU Eindhoven, 5600 MB, Eindhoven, Netherlands

* These authors contributed equally

# Corresponding author: j.tel@tue.nl

Supplementary Table 1: Eight-digit binary logic for each cell isolation unit.

	**1**	**2**	**3**	**4**	**5**	**6**	**7**	**8**
**1**	0	1	0	1	0	1	0	1
**2**	0	1	0	1	0	1	1	0
**3**	0	1	0	1	1	0	0	1
**4**	0	1	0	1	1	0	1	0
**5**	0	1	1	0	0	1	0	1
**6**	0	1	1	0	0	1	1	0
**7**	0	1	1	0	1	0	0	1
**8**	0	1	1	0	1	0	1	0
**9**	1	0	0	1	0	1	0	1
**10**	1	0	0	1	0	1	1	0
**11**	1	0	0	1	1	0	0	1
**12**	1	0	0	1	1	0	1	0
**13**	1	0	1	0	0	1	0	1
**14**	1	0	1	0	0	1	1	0
**15**	1	0	1	0	1	0	0	1
**16**	1	0	1	0	1	0	1	0

The numbers, 1 to 16, in the header column represent the cell-isolation units whereas the numbers, 1 to 8, in the header row are the respective control lines on the microfluidic chip. For the eight-digit logic, 1 means that the control line is pressurized and the membrane valves on that line are actuated closed whereas 0 means that the control line has no pressure and the membrane valves on that line are actuated open. For example, to address and open cell isolation unit 13, control lines 2, 4, 5, and 7 are actuated open whereas other control lines are actuated closed.

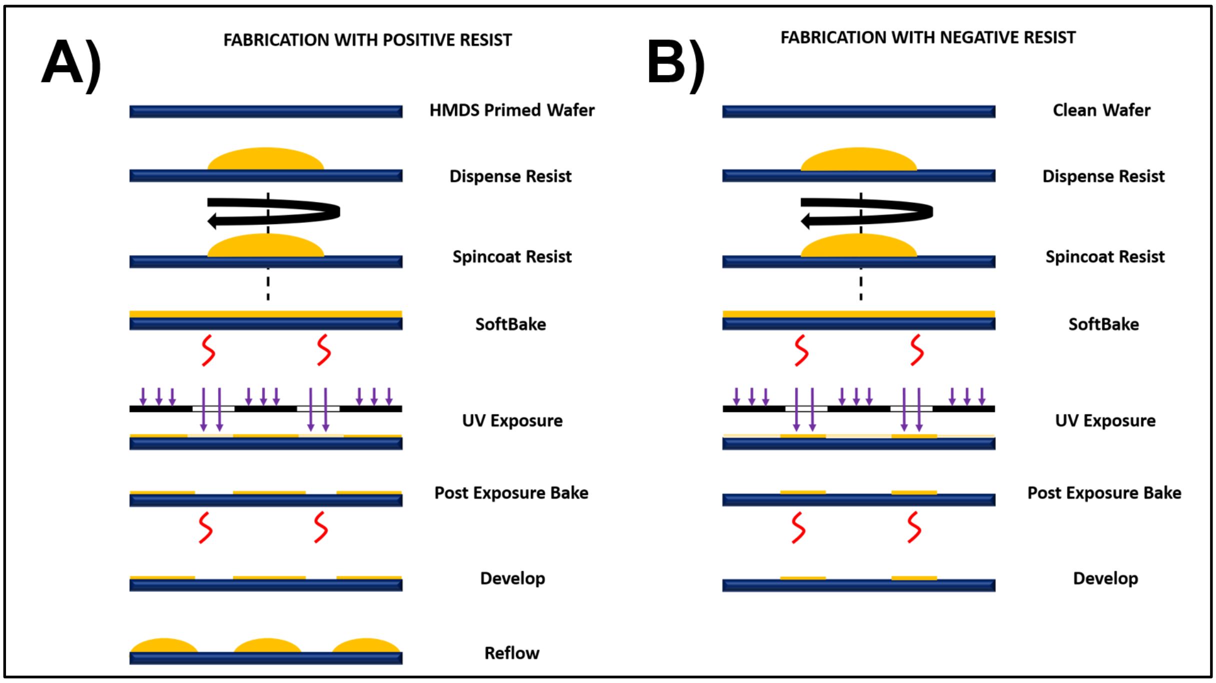

Supplementary Figure 1: Microfabrication method for channel fabrication. (A) Steps of channel fabrication when using positive resist. (B) Steps of channel fabrication when using negative resist.

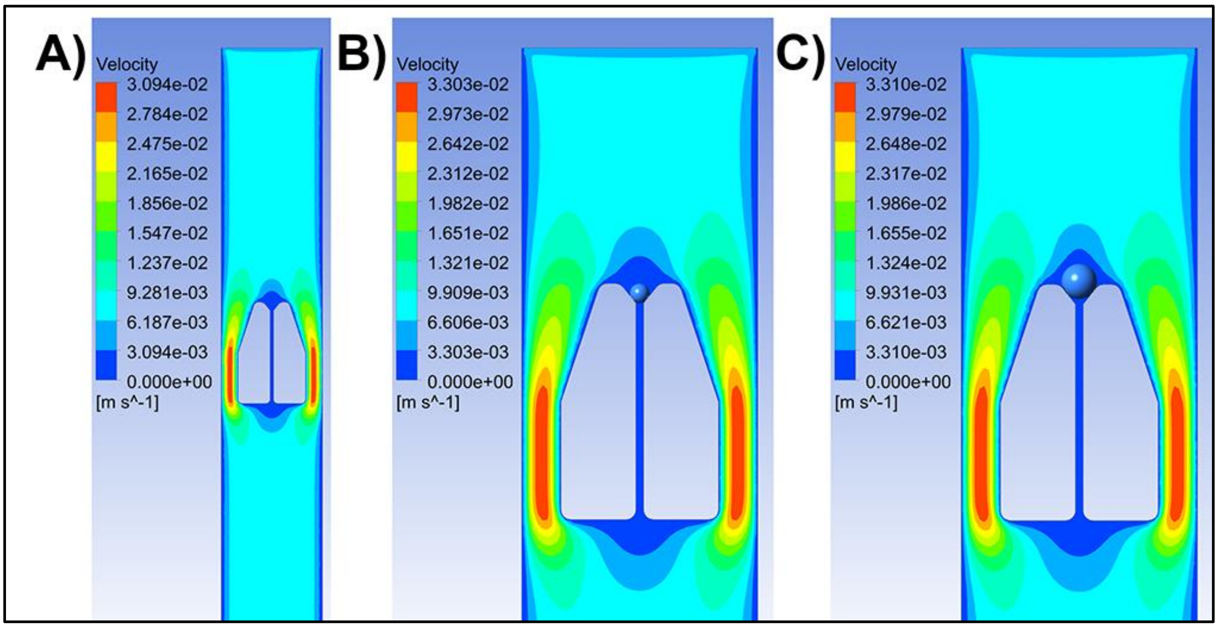

Supplementary Figure 2: Simulation results for flow profile. (A) Flow profile in an empty channel at a flow rate of 1 µL/min. (B and C) Flow profile in the channel when particles of size 10 µm (B) and 18 µm (C) are isolated by the pillars.

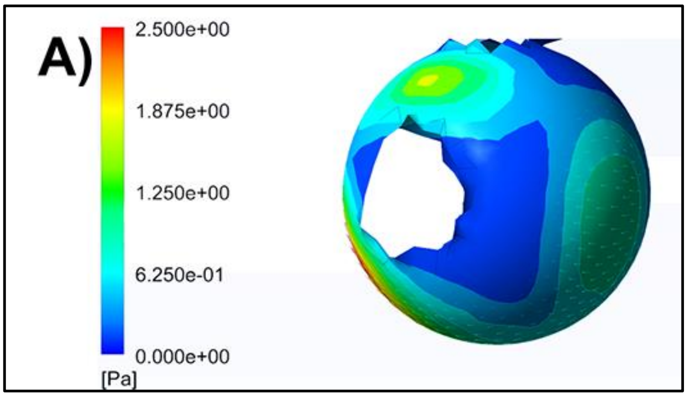

Supplementary Figure 3: Simulation result for wall stress. Under the influence of gravity the isolated cells can move to the bottom of the channel, where the experienced shear stress, by virtue of flow of 1 µL/min, ranges between 0.5 Pa and 2.5 Pa.

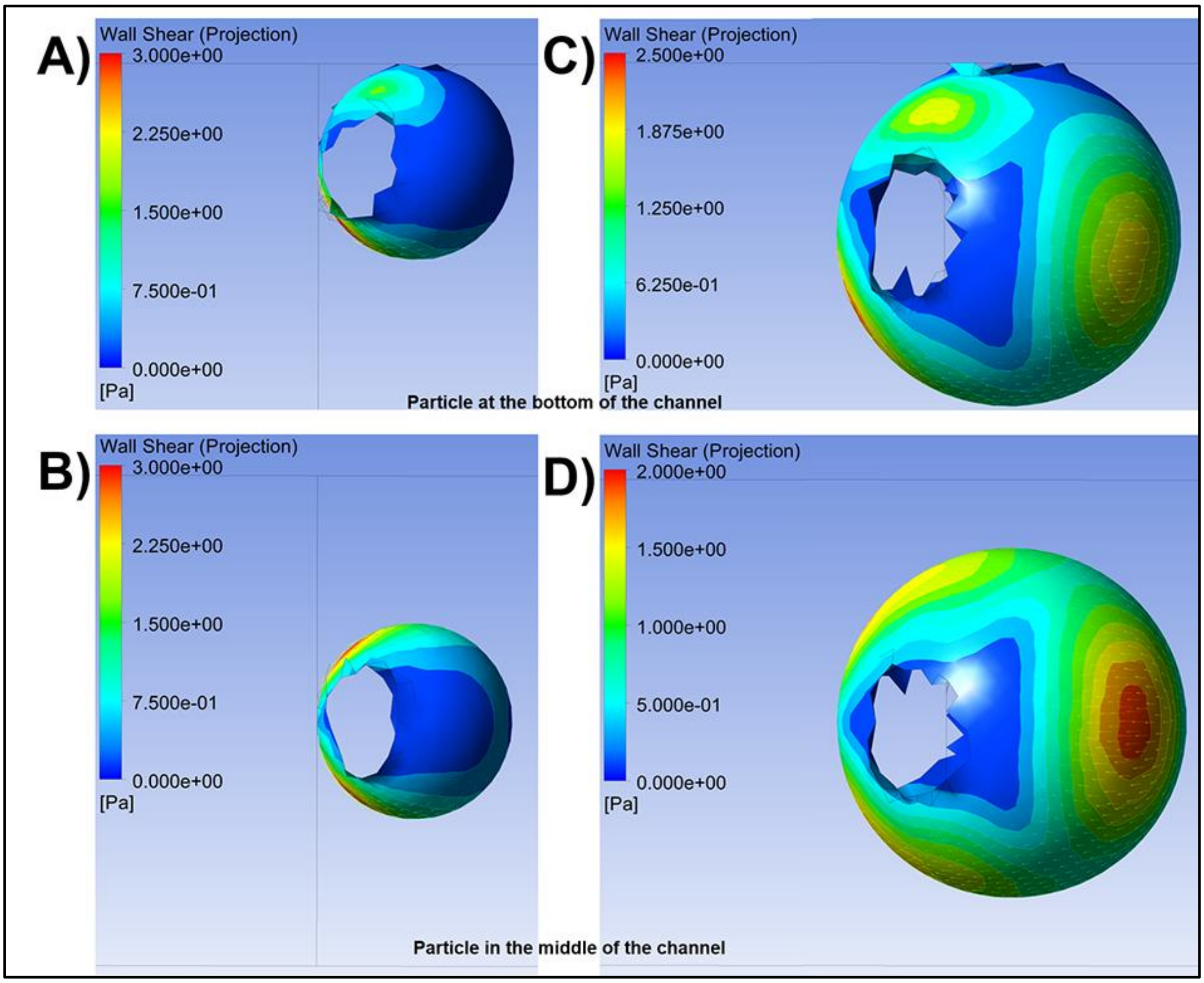

Supplementary Figure 4: Simulation results for wall shear for different particle size. Shear stress profile on particles of size 10 µm (A, B) and 18 µm (C, D) when subjected to flow rate of 1 µL/min.

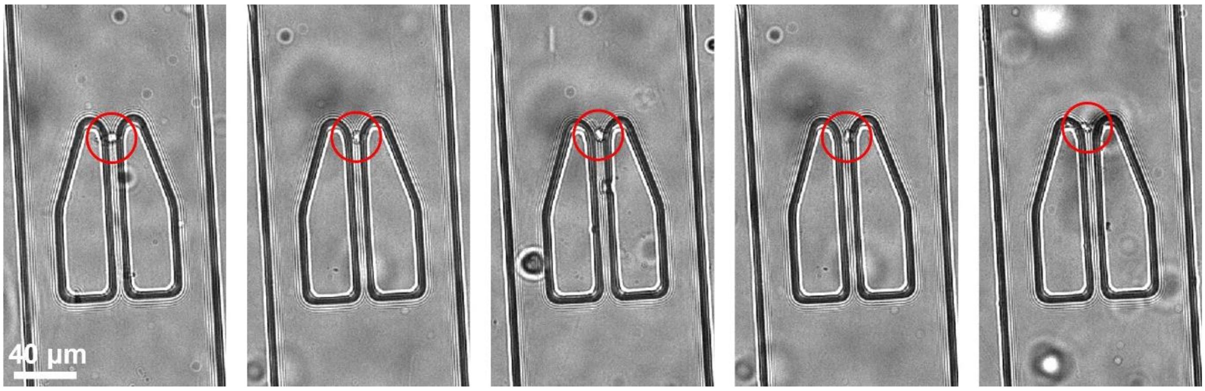

Supplementary Figure 5: Single cell trapping efficiency for primary cells. Trapping of single primary cells from peripheral blood mononuclear cell (PBMC) sample.

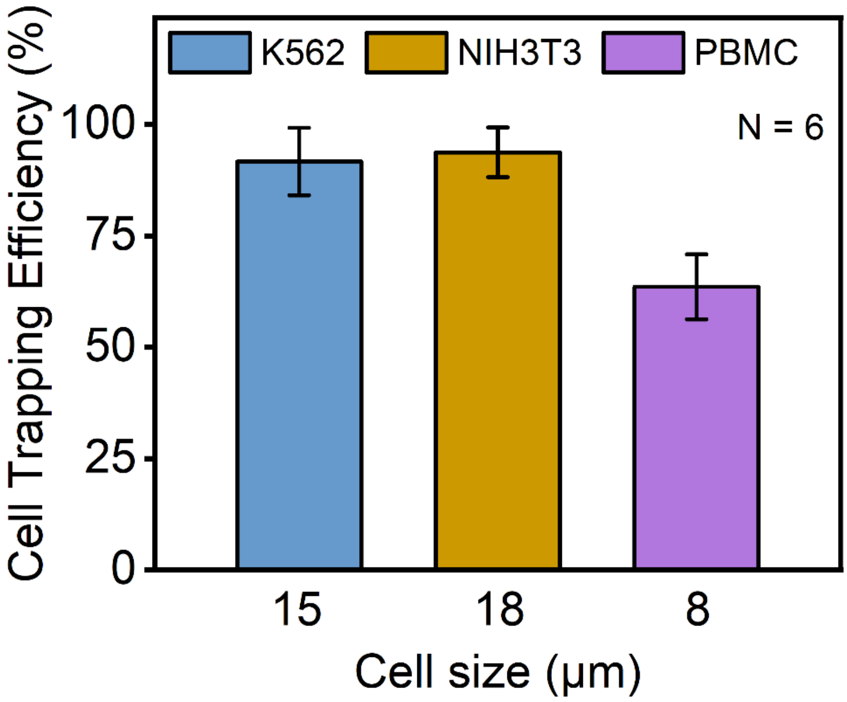

Supplementary Figure 6: Cell trapping efficiency as a function of cell size. Trapping efficiency for different cell types in relatively large (K562 and NIH3T3) and small (PBMC) size. Error bar represents mean +/- SD.

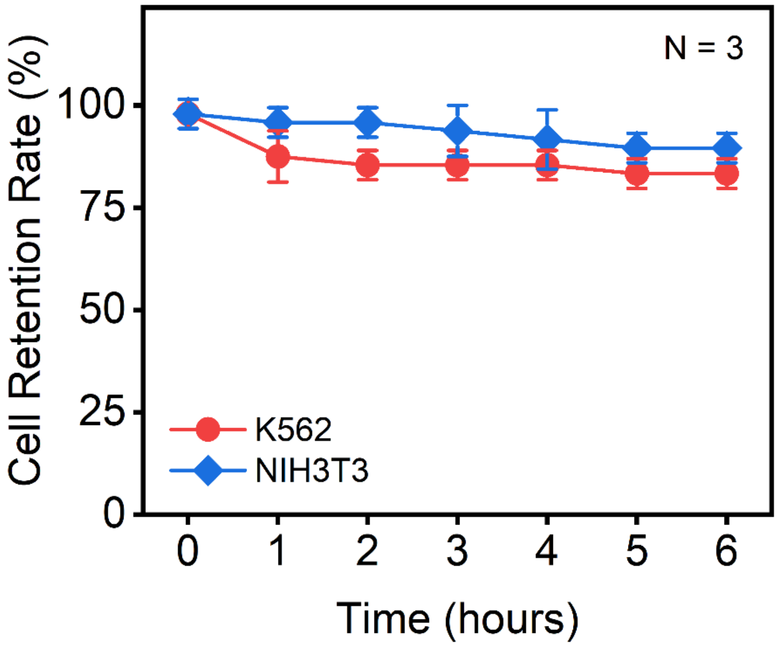

Supplementary Figure 7: Retention rate in channels. Retention rate of single cells in the microfluidic channels with over 85% cells being retained up and until 6 hours. The retention rate of NIH3T3 is higher than K562 cells given their adherent nature. Error bar represents mean +/- SD.

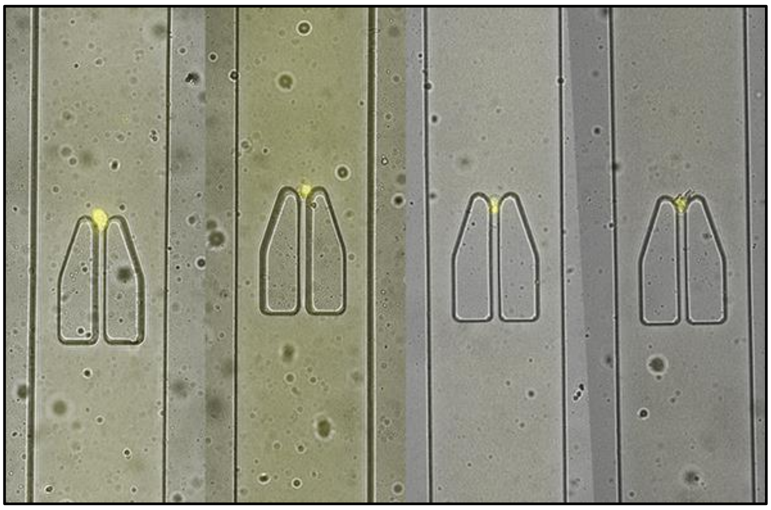

Supplementary Figure 8: Live/dead cell identification. Dead K562 cells identified at time, t = 0.

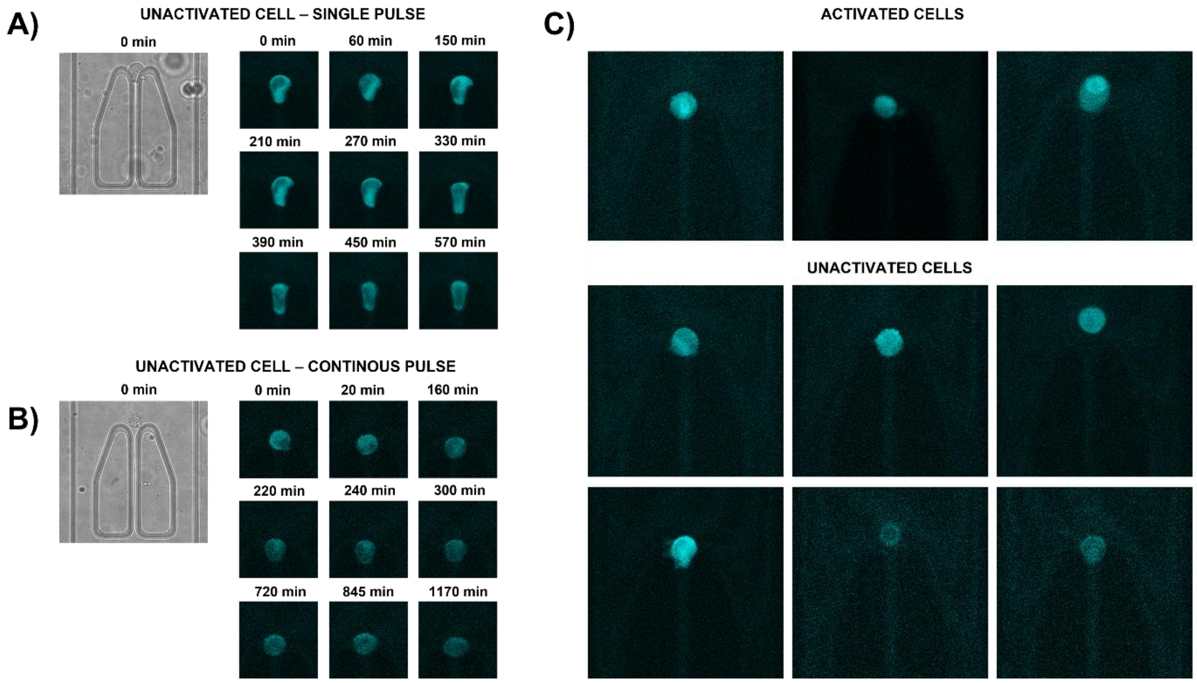

Supplementary Figure 9: Nuclear translocation of STAT-1 protein. (A,B) Representative fluorescence images of NIH3T3 cells that showed no STAT-1 activity when treated with pulsatile and continuous stimulation patterns. (C) Representative results of active and unactive cells when stimulated with single pulse of 10 minutes.
