# Supplementary material for: Microfluidic chip for precise trapping of single cells and temporal analysis of signaling dynamics
Source: Commun Eng. 2022 Jul 25;1:18. doi: 10.1038/s44172-022-00019-2 (PMC10955935; doi:10.1038/s44172-022-00019-2)
Supplement: Supplementary file 1 — Tel_PR file [file 44172_2022_19_MOESM1_ESM.pdf]

nature portfolio

Peer Review File

Microfluidic chip for precise trapping of single cells and temporal analysis of signaling dynamics

Open Access This file is licensed under a Creative Commons Attribution 4.0 International License, which permits use, sharing, adaptation, distribution and reproduction in any medium or format, as long as you give appropriate credit to the original author(s) and the source, provide a link to the Creative Commons license, and indicate if changes were made. In the cases where the authors are anonymous, such as is the case for the reports of anonymous peer reviewers, author attribution should be to 'Anonymous Referee' followed by a clear attribution to the source work. The images or other third party material in this file are included in the article's Creative Commons license, unless indicated otherwise in a credit line to the material. If material is not included in the article's Creative Commons license and your intended use is not permitted by statutory regulation or exceeds the permitted use, you will need to obtain permission directly from the copyright holder. To view a copy of this license, visit http://creativecommons.org/licenses/by/4.0/.

Reviewers' comments:

Reviewer #1 (Remarks to the Author):

The work authored by Sinha et al. presents a set of microfluidic large-scale integration (mLSI) chips that allow biological researchers to implement complex biological assays at single-cell resolutions. The manuscript is well prepared with sufficient figures to elaborate the working mechanism, and demonstration of their success in differentiating population cells in response to external stimuli. The work is based on the longstanding establishment of pneumatic valves/traps in microfluidics, single-cell trapping methods, parallel manipulation of fluids, etc. Before being accepted for publication, I would recommend the authors to further prove/demonstrate two major points:
1) The feasibility of this system in the manipulation of primary tissue-derived cells, when the extract population is not entirely composed of single-cell suspension, but irregular single cells and cell clusters with a higher gluing ability (i.e. more "sticky"). The responses of primary cells toward stimuli, e.g. drug administration, has straightforward implication on biology and medicine, rather than cell lines. However, non-homogeneous primary cell suspension would have distinctive flow behaviors as cell line suspensions presented in this study. Whether the "single-cell trap" has equivalent performance remains elusive.
2) Figure 6E and 6F show the drastic difference in presenting individual cells' response toward stimuli between mLSI single cells and bulk cells. However, it remains unknown of the ground truth, thus cannot reach any decision of the reliability of mLSI single-cell examination. It should be compared with a well-recognized technical report. (line 327-328)
3) A minor: the figure 3D is difficult to read. The meaning of the irregular, sharp-edged hole is not properly explained.

Reviewer #2 (Remarks to the Author):

The paper introduced an integrated microfluidic chip for 16 channel single-cell regulation and applied them for analysis of cell signaling dynamics. Similar types of single-cell regulation chips have been reported many years ago after the creation of the Quake pump. The novel part of the research may be the application of such a system for single-cell analysis. But there are several suggestions for the authors to improve the paper before it can be published.
1. In the introduction part, it is valuable to add some background for the three types of single-cell regulation modes in microfluidic devices, including those applying Quake pump, hydrodynamically formed arrays, and the droplets. The pros and cons of these modes should be compared so that the readers can better understand why the authors choose the first type for single-cell analysis here.
2. The references cited contain many review papers rather than the work of the first contributors. It is better to follow the ethic to give credit to the first contributors, and the ones who set the milestones. Also, the names of the authors are cited with "et al", which is uncommon and unclear for the readers. Besides, some of the famous papers in the field are not cited, e.g., the works of Yanyi Huang from Peking University, which might be misleading for the novices to this field.
3. For chip design, it is not clear whether it is novel or not. The authors claimed it is "inspired from previous designs (Ref. 19, 23, and 24)", it is necessary to tell what the similarities with the former ones are, and what their differences are. If there is any improvement in this work than the former ones? Clarifying these points helps the readers to better understand the advantage of the proposed device.
4. There are also previous works utilizing similar single-cell trapping barriers, but the trapping units are physically connected, these works should be mentioned and compared.
There are also some minor comments:
1. Some typos exist in the paper, please carefully revise them.
2. The notes in Figures 1D and1 F are much smaller than those in 1B, 1C, and 1E. Some of the words are in capital form some are not. Please carefully revise them.

3. Scale bars are necessary for all the images and designs in the figures.

Reviewer #3 (Remarks to the Author):

This manuscript presents a microfluidic device for single-cell level trapping and biosensing. The work is interesting, the entire manuscript is well written and organized. The experiment is well-designed, and the quality of the presentation is high. However, the major issue of this work is the technical novelty and system performance in real high throughput biological assays. I would like to recommend that this work be published in Communications Engineering after my concerns are addressed. The detailed comments are listed below:

Major comments
1. Microfluidic technique aspect, what is the major novelty of this work? The Quake valving system is introduced around 20 years ago. The trapping method utilizing the same kind of hydrodynamic mechanism had been well developed and reported (https://www.pnas.org/doi/10.1073/pnas.1510328112; https://doi.org/10.1016/bs.mcb.2018.09.010; https://doi.org/10.1364/BOE.402526; https://doi.org/10.1155/2014/929163). A more comprehensive literature review focusing on the microfluidic trapping technique is needed to discuss the state-of-art in the field. What is the new and better of your mechanism? Why it is better than a well-array system and micro trenches that provide much higher throughput and better stability at static status.
2. An investigation of the relationship between cell size and/or surface stiffness vs the gap between pillars is needed.
3. Trapping efficiency, to guarantee at least one cell to be trapped in the pillar structure, how many cells in total are needed to flow through the channel and how long does the trapping processing cost? if you need to load lots of cells to archive the so-called 90% trapping efficiency, then this method is not the high efficient and high-throughput way.
4. How to hold the cell in trapping when stopping flow if we need to stop the current flow and introduce another flow for the second treatment. For example, a digital ELISA requires multiple washing.
5. For the simulation aspect, a mesh refinement assay is needed to ensure the accuracy of the simulation results.
6. During the biological assays, the treatment reagents are injected into channels continuously. Would any fluctuation in flow speed or flow rate cause loss of cell trapping?
Minor Comments

1. Scale bars are missing in most figures (Figures 2, 4,5, and 6)

Reviewer #1 (Remarks to the Author):

The work authored by Sinha et al. presents a set of microfluidic large-scale integration (mLSI) chips that allow biological researchers to implement complex biological assays at single-cell resolutions. The manuscript is well prepared with sufficient figures to elaborate the working mechanism, and demonstration of their success in differentiating population cells in response to external stimuli. The work is based on the longstanding establishment of pneumatic valves/traps in microfluidics, single-cell trapping methods, parallel manipulation of fluids, etc. Before being accepted for publication, I would recommend the authors to further prove/demonstrate two major points:

The authors would like to thank the reviewer for the kind words and constructive comments to improve the current manuscript for publication. As suggested by the reviewer, we have addressed the individual comments and have provided the explanation below.

1) The feasibility of this system in the manipulation of primary tissue-derived cells, when the extract population is not entirely composed of single-cell suspension, but irregular single cells and cell clusters with a higher gluing ability (i.e. more "sticky"). The responses of primary cells toward stimuli, e.g. drug administration, has straightforward implication on biology and medicine, rather than cell lines. However, non-homogeneous primary cell suspension would have distinctive flow behaviors as cell line suspensions presented in this study. Whether the "single-cell trap" has equivalent performance remains elusive.

We appreciate that the reviewer brought up the feasibility of using this device with primary cells that are more irregular in suspension and also have higher gluing ability. In fact, sometimes these cells are also rare in number and seeding them may also result in cell loss, giving us results that are different from the ones obtained with the more regular suspension cell line. We repeated the cell trapping with primary cells from peripheral blood mononuclear cell (PBMC) sample, to observe that while we were able to retain the cells in the traps, the efficiency was indeed lower (new supplementary figure – Figure S5). However, we do emphasize that these trap designs can easily be modified to cater to the variability in size on primary cells and ensure higher trapping efficiency. We have discussed these findings in the results section and included the additional figure in the supplementary section.

2) Figure 6E and 6F show the drastic difference in presenting individual cells' response toward stimuli between mLSI single cells and bulk cells. However, it remains unknown of the ground truth, thus cannot reach any decision of the reliability of mLSI single-cell examination. It should be compared with a well-recognized technical report. (line 327-328)

We do understand the concerns of the reviewer concerning the reliability of the mLSI based single-cell experimentation. Previously, complex mLSI chip designs have been used to comprehend the transcription factor activities of NF-κB (https://doi.org/10.1016/j.celrep.2016.03.033) that showed digital switch like response under certain input conditions.

Similar to this transcription factor, we hypothesized that STAT could also have distinct nuclear translocation pattern when treated with interferons either continuously or under pulsed conditions. In our immune system, not all cells receive and process the stimuli in the same order and often have received stimuli in pulses, variable as a function of both time and doses. With the intent to understand whether STAT also follows a similar pattern, we chose the mLSI design as the best alternative. It allows us to deliver pulses of stimuli and also by integrating pillar like traps we could perform these investigations at single cell level, that allowed us to observe the behaviour in single cells. This kind of information is also often lost in bulk studies, wherein the scope of manual error also intensifies.

With thoughts along the similar hypothesis, we aimed to investigate if time modulated activation of an apoptotic pathway will reveal distinct response patterns during apoptotic induction, which was evident in our results. This is also useful for future studies to check the response of drug-resistant and drug-responsive tumor cells against drugs that are not delivered continuously but in pulses. The intention of using mLSI was to vary the stimulation patterns and analyse the behavior in individual cells and this is where mLSI platform holds relevance. We indeed have cited some important papers from this line of research as a part of our motivation for this study.

3) A minor: the figure 3D is difficult to read. The meaning of the irregular, sharp-edged hole is not properly explained.

The authors would like to thank the reviewer for this valid point. We have explained in the figure legend as to why the holes appears. The explanation is provided as follows: ‘The white space in the particle, that can be seen as hole, is the part of the section that coincides attaches with the trap during assembly. Upon hiding the traps, that specific section in the rigid particles also disappears giving the impression of hole. This section and is not affected by the flow, according to the simulation software.’ We have tried other ways to resolve this issue, however, the software did not permit so, unfortunately, and this is the best representation we can provide. Also, with the clarity of the figure. While the colour schemes represent the shear stress values, the arrows in white are the direction of stress, in the direction of flow. We have used the most optimal setting to represent this figure.

Reviewer #2 (Remarks to the Author):

The paper introduced an integrated microfluidic chip for 16 channel single-cell regulation and applied them for analysis of cell signaling dynamics. Similar types of single-cell regulation chips have been reported many years ago after the creation of the Quake pump. The novel part of the research may be the application of such a system for single-cell analysis. But there are several suggestions for the authors to improve the paper before it can be published.

The authors are thankful to the reviewer for their constructive and considerate feedback. Based on the suggestions, we have worked on the comments suggested by the reviewer towards improving the manuscript.

1. In the introduction part, it is valuable to add some background for the three types of single-cell regulation modes in microfluidic devices, including those applying Quake pump, hydrodynamically formed arrays, and the droplets. The pros and cons of these modes should be compared so that the readers can better understand why the authors choose the first type for single-cell analysis here.

We appreciate reviewer's suggestion to discuss three different types of single-cell microfluidic platforms along with their advantages and disadvantages that led to the selection of mLSI designs for this investigation. Further we have also cited a previously published paper from our group that discusses in detail these three microfluidic platforms (https://doi.org/10.3389/fimmu.2018.02373). We included this in the Introduction section of the manuscript.

2. The references cited contain many review papers rather than the work of the first contributors. It is better to follow the ethic to give credit to the first contributors, and the ones who set the milestones. Also, the names of the authors are cited with "et al", which is uncommon and unclear for the readers. Besides, some of the famous papers in the field are not cited, e.g., the works of Yanyi Huang from Peking University, which might be misleading for the novices to this field.

We agree with the reviewer to refer to pioneers in the field and the first papers rather than citing review papers that we have now updated in the modified manuscript. In reference to citing with 'et al' we use the standard and widely accepted Mendeley software set to Nature citation style for this manuscript. Mendeley automatically formats the citation style and unfortunately we do not have much control over this. We have also updated our citations and reference with more famous papers in this research area.

3. For chip design, it is not clear whether it is novel or not. The authors claimed it is "inspired from previous designs (Ref. 19, 23, and 24)", it is necessary to tell what the similarities with the former

ones are, and what their differences are. If there is any improvement in this work than the former ones? Clarifying these points helps the readers to better understand the advantage of the proposed device.

We agree with the reviewer that it will be useful for future readers to understand the differences between our current design and previously published work. This will help them realise the advantages of this design and adapt it to their laboratory setting. We have incorporated this in the results and discussion section 'Two-Layer Microfluidic Design for Automated Delivery of Pulsatile Stimuli Patterns, On-Chip'. In the updated version of the manuscript these reference numbers have been updated to 19, 32, and 33.

4. There are also previous works utilizing similar single-cell trapping barriers, but the trapping units are physically connected, these works should be mentioned and compared.

We thank the reviewer for providing us with this suggestion that helped us to improve the previous version of the manuscript. We have included a brief discussion in the results section, 'Hydrodynamic Physical Trapping Mechanism for Efficient Trapping of Single-Cells, On-Chip' wherein we have referenced to some previously published similar work and discussed the benefits and drawbacks as a whole.

There are also some minor comments:

1. Some typos exist in the paper, please carefully revise them.

We have carefully proofread the manuscript to check for errors and inconsistencies.

2. The notes in Figures 1D and1 F are much smaller than those in 1B, 1C, and 1E. Some of the words are in capital form some are not. Please carefully revise them.

We apologise for the inconsistency in Figure 1. In the new figures, the font size appear to be more consistent. Additionally, some words are capitalized as they are abbreviated forms of proteins/chemicals/molecules. There were additional words in capitalized form that we have now changed.

3. Scale bars are necessary for all the images and designs in the figures.

We would like to thank the reviewer for highlighting the missing scale bars. We have now updated our figures with scale bars.

Reviewer #3 (Remarks to the Author):

This manuscript presents a microfluidic device for single-cell level trapping and biosensing. The work is interesting, the entire manuscript is well written and organized. The experiment is well-designed, and the quality of the presentation is high. However, the major issue of this work is the technical novelty and system performance in real high throughput biological assays. I would like to recommend that this work be published in Communications Engineering after my concerns are addressed. The detailed comments are listed below:

We would like to thank the reviewer for their kind words and appreciate their feedback, which we have addressed in the current version of the manuscript. We also agree with the reviewer that this platform does not offer high throughput. Achieving really high throughput with mLSI platforms, as compared to other microfluidic approaches, remains challenging. However, we trade this feature off with the ability to provide greater control over cellular microenvironment. We have addressed and included this relevant point in our updated manuscript, in additional to the other suggestions made by the reviewer.

Major comments

1. Microfluidic technique aspect, what is the major novelty of this work? The Quake valving system is introduced around 20 years ago. The trapping method utilizing the same kind of hydrodynamic mechanism had been well developed and reported (https://www.pnas.org/doi/10.1073/pnas.1510328112; https://doi.org/10.1016/bs.mcb.2018.09.010; https://doi.org/10.1364/BOE.402526; https://doi.org/10.1155/2014/929163). A more comprehensive literature review focusing on the microfluidic trapping technique is needed to discuss the state-of-art in the field. What is the new and better of your mechanism? Why it is better than a well-array system and micro trenches that provide much higher throughput and better stability at static status.

We appreciate reviewer's feedback to incorporate a comparative reasoning as to why our system is better from different aspects. We have referenced to more work than done previously in this new version of manuscript and also discussed briefly the pros and cons of the previously published work in comparison to our design. This has been incorporated in the results section: 1. Two-Layer Microfluidic Design for Automated Delivery of Pulsatile Stimuli Patterns, On-Chip and 2. Hydrodynamic Physical Trapping Mechanism for Efficient Trapping of Single-Cells, On-Chip.

2. An investigation of the relationship between cell size and/or surface stiffness vs the gap between pillars is needed.

We thank the reviewer for the suggestion and understand that this analysis will provide a better insight into how cell size or stiffness will influence the efficiency of trapping given the gap between the pillars. While given the current limitation with resources, we were only able to assess the relationship between the cell size and the gap between the pillars to demonstrate the trapping efficiency. We have provided an additional figure in our supplementary section, Figure S6, wherein we show that with decrease in cell size (primary cells from PBMCs which are much smaller in size compared to the cell lines) the trapping efficiency also reduces. Given the smaller size and perhaps also higher elasticity, the cells can squeeze through this small gap of 4 microns, resulting in reduced trapping efficiency. However, if we further decrease the this gap between the pillars, we will be able to retain these cells. For cells smaller than this, we might require further design modification. However, for larger cells size, greater than 15 microns, we worked well with the current design.

3. Trapping efficiency, to guarantee at least one cell to be trapped in the pillar structure, how many cells in total are needed to flow through the channel and how long does the trapping processing cost? if you need to load lots of cells to archive the so-called 90% trapping efficiency, then this method is not the high efficient and high-throughput way.

We thank the reviewer for highlighting the potential issues with our trapping mechanism. Based on our observations and experimental trials we indeed do not require a lot of cells flowing through the channel to ensure the trapping of single cells. With the cell loading density and optimal pressure/flow rate values, we are able to guarantee the trapping of single cells with very low number of cells flowing through the channel. Using the parameters described, the cell density in the flow channel is low but we are able to trap the cell as early as the first cell that flows through the channel. If this does not work, we require additional 2-3 cells but within this limit, we have managed to successfully trap the cells.

4. How to hold the cell in trapping when stopping flow if we need to stop the current flow and introduce another flow for the second treatment. For example, a digital ELISA requires multiple washing.

This is indeed an interesting point to raise with our device design, which we have also thought about during the design and fabrication process. The traps are efficient to hold the cells in position as long as we maintain the determined values for pressure and flow rates, as mentioned in the manuscript, during the exchange of media and other reagents. However, under unforeseen circumstances the cells do have the possibility to be washed away, as shown in Figure S7. But over 80% of single cells retained in channels for over 20 hours with medium exchange.

5. For the simulation aspect, a mesh refinement assay is needed to ensure the accuracy of the simulation results.

We do understand reviewer's concern with accuracy of the simulation results that can be enhanced with further meshing. In our simulation model, we have used the finest mesh available with the software to obtain the results presented in the manuscript. Furthermore, what we obtained in our simulation model is similar to what we observed in our experimental findings. We have previously tried different mesh types and sizes to select the most results that gave the most accurate results, presented in this manuscript.

6. During the biological assays, the treatment reagents are injected into channels continuously. Would any fluctuation in flow speed or flow rate cause loss of cell trapping?

As shown in Figure S7, over 80% of single cells retained in channels for over 20 hours with medium exchange. With continuous flushing, fluctuation is much smaller than medium exchange (switching flow). So it can be sure that at least over 80% of single cells retained in channels for over 20 hours under continuous flushing.

Minor Comments

1. Scale bars are missing in most figures (Figures 2, 4,5, and 6)

We thank the reviewer for their suggestions and have updated the given figures with the scale bars.

REVIEWERS' COMMENTS:

Reviewer #2 (Remarks to the Author):

The paper was improved, and it is now recommended for publication.

Reviewer #3 (Remarks to the Author):

The feedback from authors have address my concerns. I would like to recommend this work to be published on Commuications Engineering after carefully proofreading
